# Supplementary figures and images for: The carbon starvation-inducible lipoprotein (Slp) influences differential adherence of Escherichia coli O157:H7 at the bovine rectoanal junction
Source: PLoS Pathog. 2026 May 18;22(5):e1013584. doi: 10.1371/journal.ppat.1013584 (PMC13193606; doi:10.1371/journal.ppat.1013584)

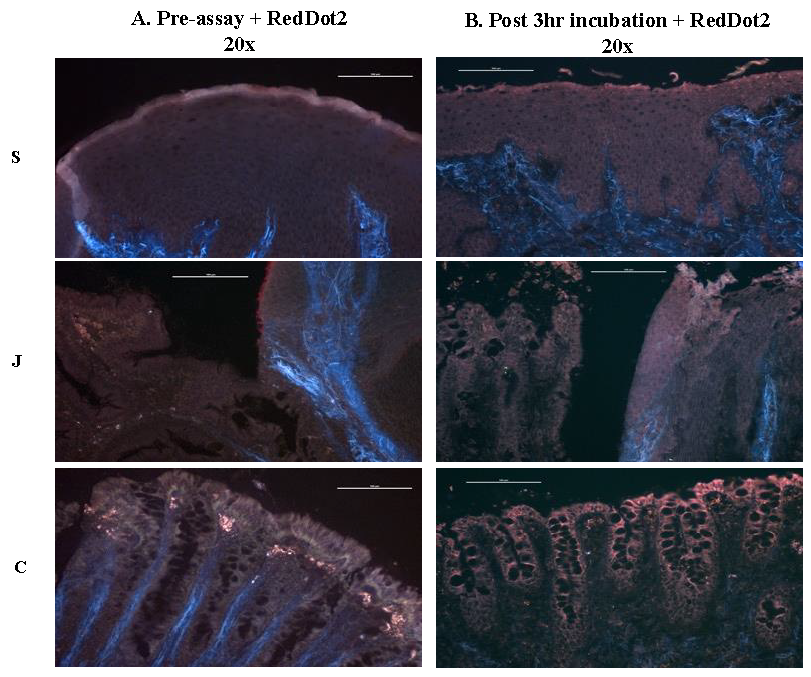

Supplement: S5 Fig — The un-fixed RAJ-IVOC tissue was stained with RedDot2 dye, (A) pre-assay and (B) post-3 h incubation. The squamous (S), junction (J), and columnar (C) regions of the stained RAJ-IVOC tissue are shown, along with the 100 µm scale bar. Images were captured at 200x magnification; the objective used is indicated on the images. The RedDot2 dye stains nuclei red in tissues with altered integrity. No red coloration of the nuclei, in both samples, reflects good tissue integrity and viability. (TIF) [file ppat.1013584.s005.tif]

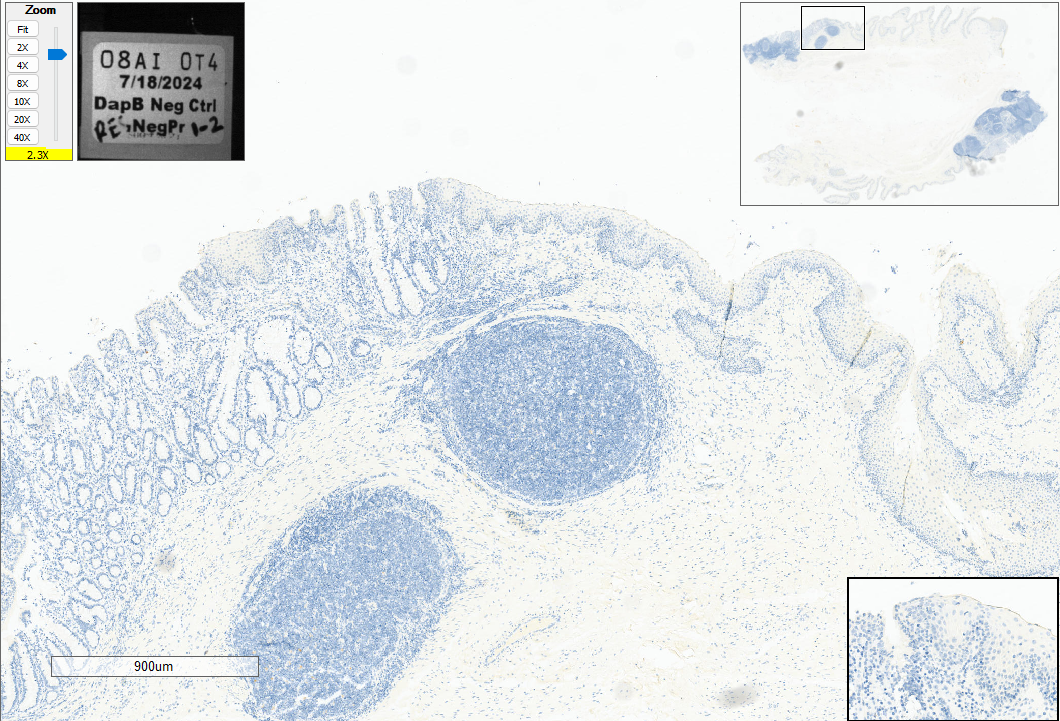

Supplement: S2 Data — (ZIP) [file ppat.1013584.s011.zip › S2_Data/Test/DapB_1-2.png]

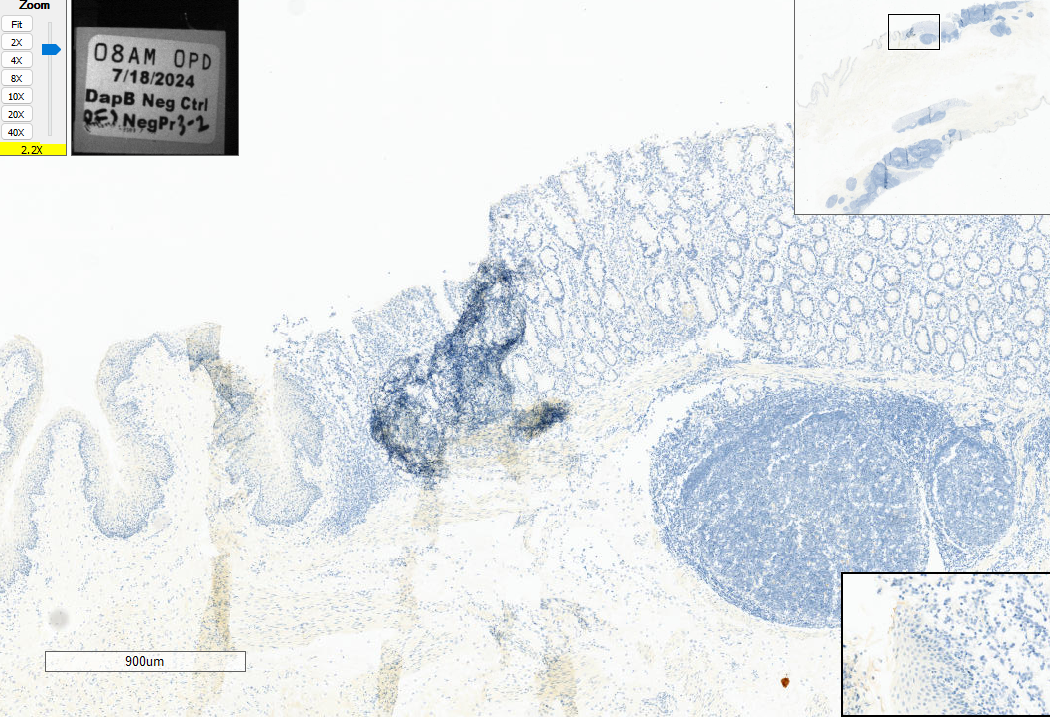

Supplement: S2 Data — (ZIP) [file ppat.1013584.s011.zip › S2_Data/Test/DapB_3-2.png]

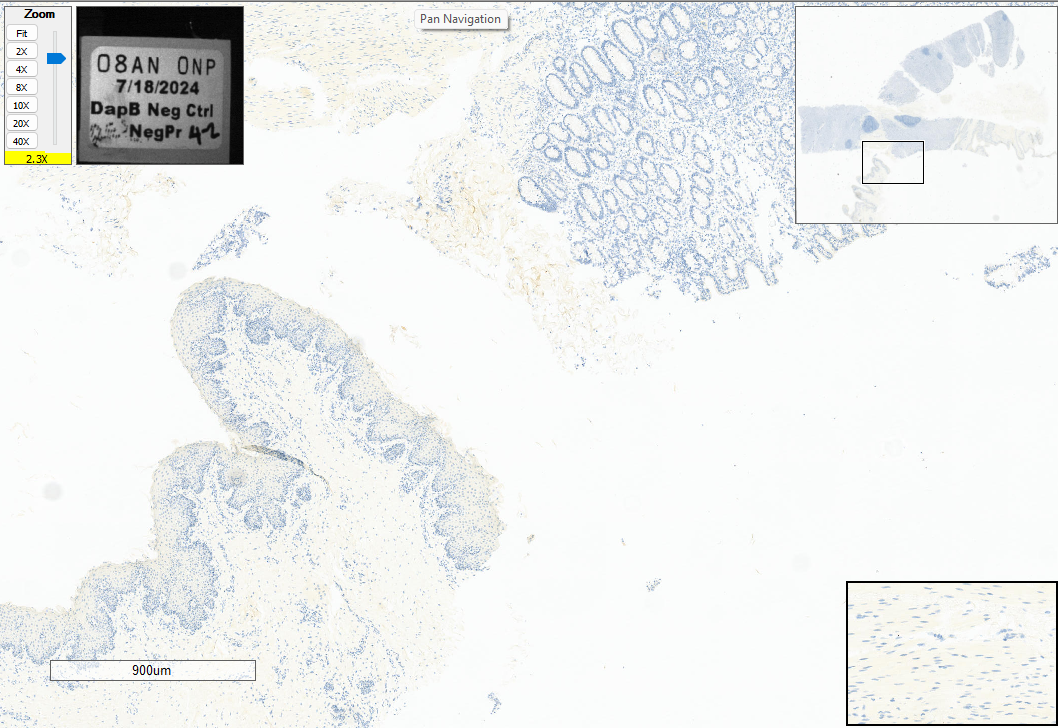

Supplement: S2 Data — (ZIP) [file ppat.1013584.s011.zip › S2_Data/Test/DapB_4-2.png]

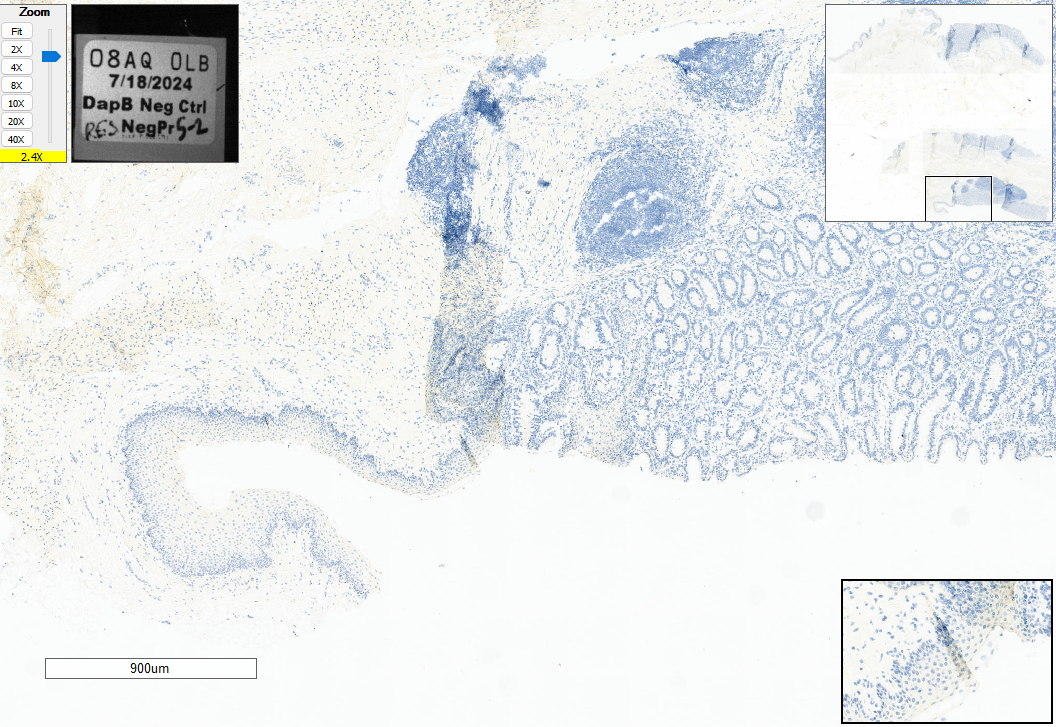

Supplement: S2 Data — (ZIP) [file ppat.1013584.s011.zip › S2_Data/Test/DapB_5-2.png]

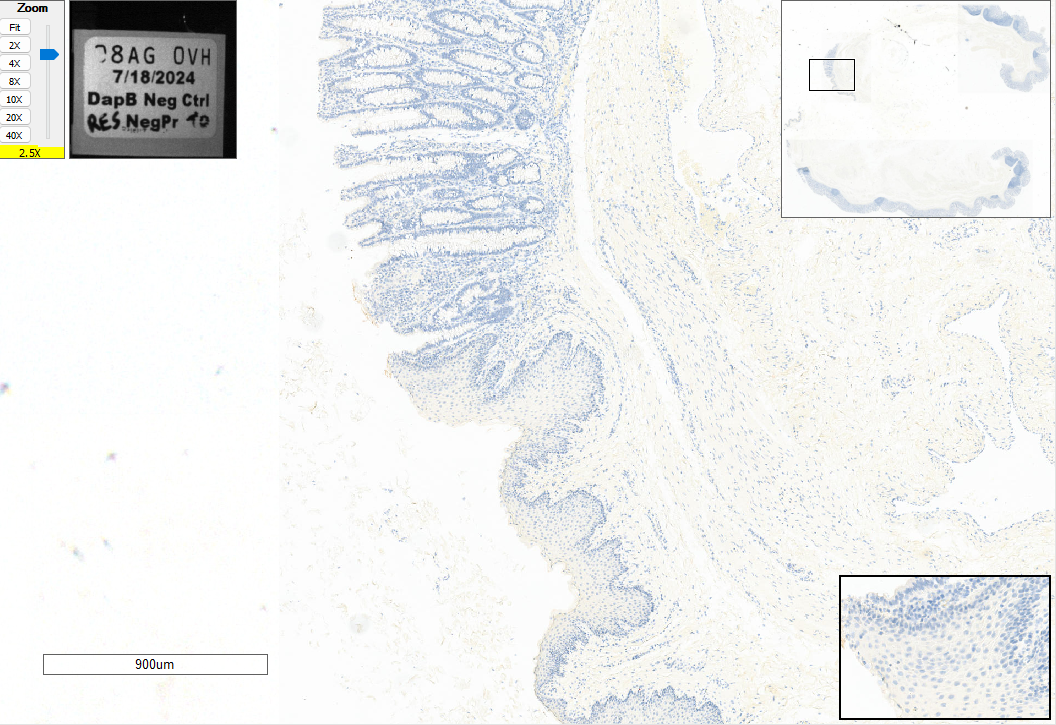

Supplement: S2 Data — (ZIP) [file ppat.1013584.s011.zip › S2_Data/Test/DapB_T0.png]

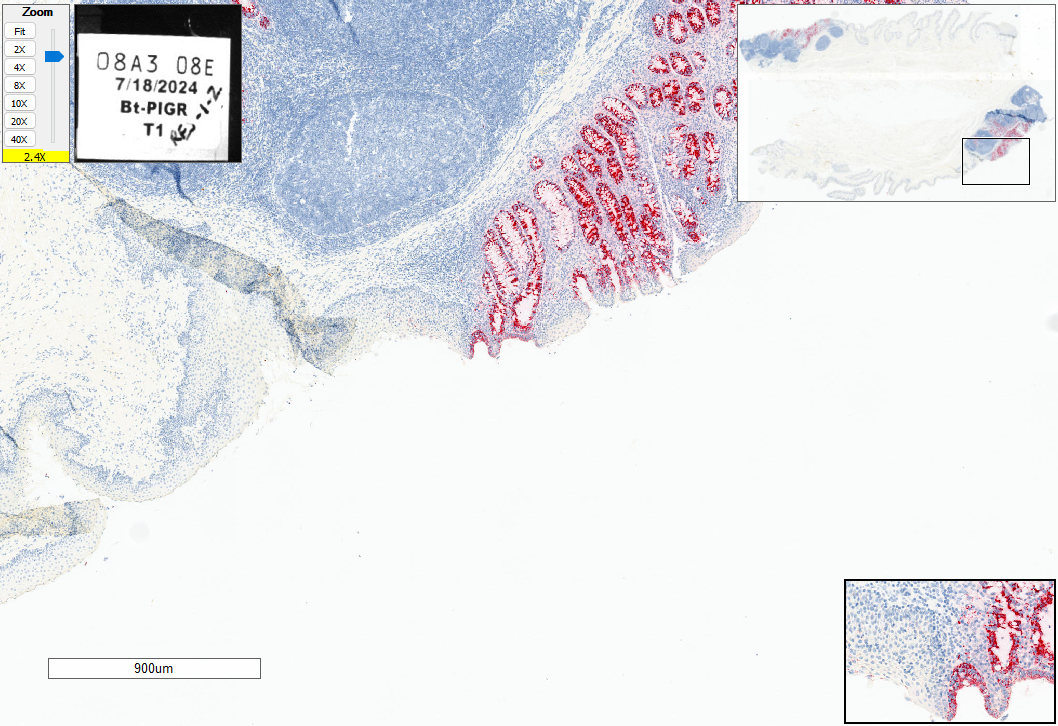

Supplement: S2 Data — (ZIP) [file ppat.1013584.s011.zip › S2_Data/Test/PIGR_1-2.png]

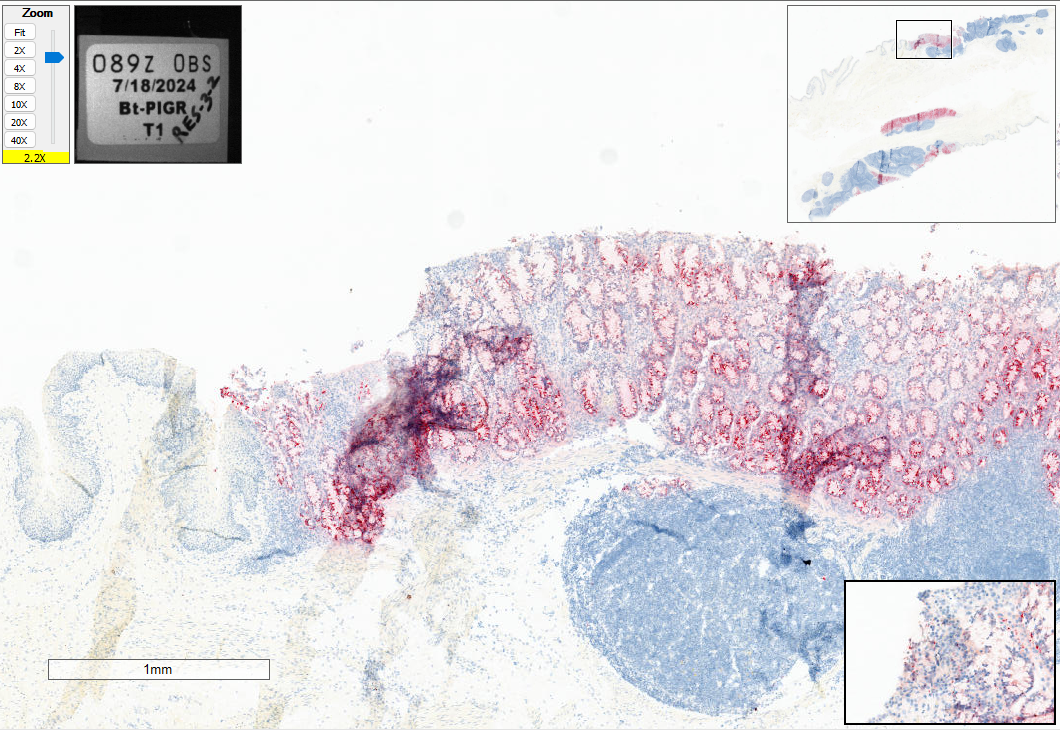

Supplement: S2 Data — (ZIP) [file ppat.1013584.s011.zip › S2_Data/Test/PIGR_3-2.png]

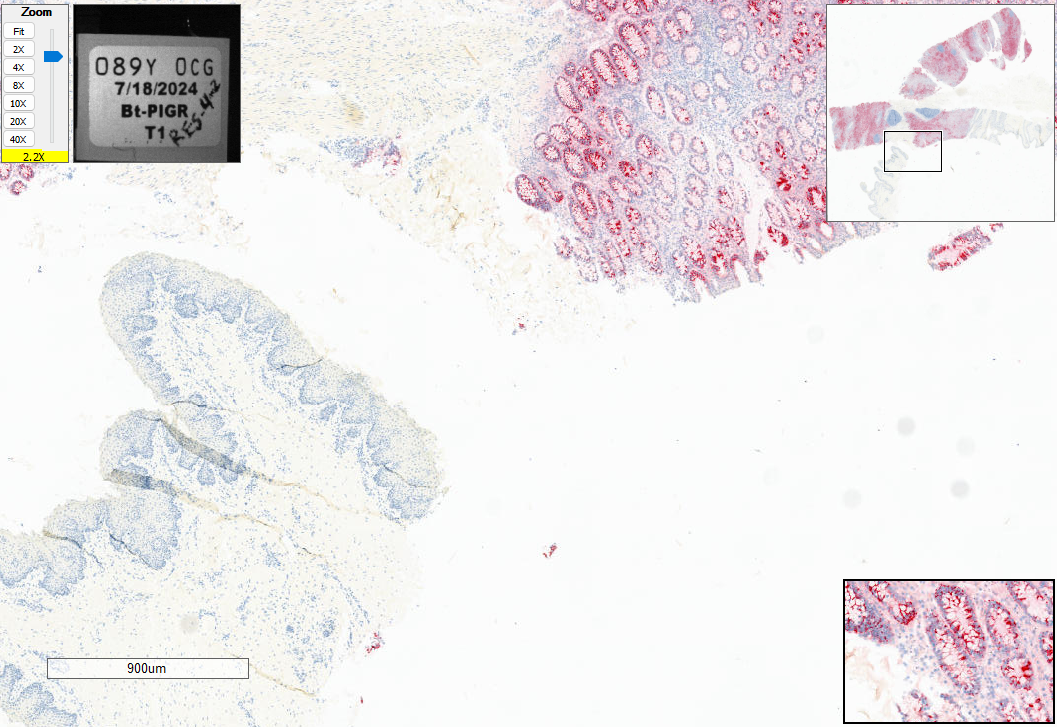

Supplement: S2 Data — (ZIP) [file ppat.1013584.s011.zip › S2_Data/Test/PIGR_4-2.png]

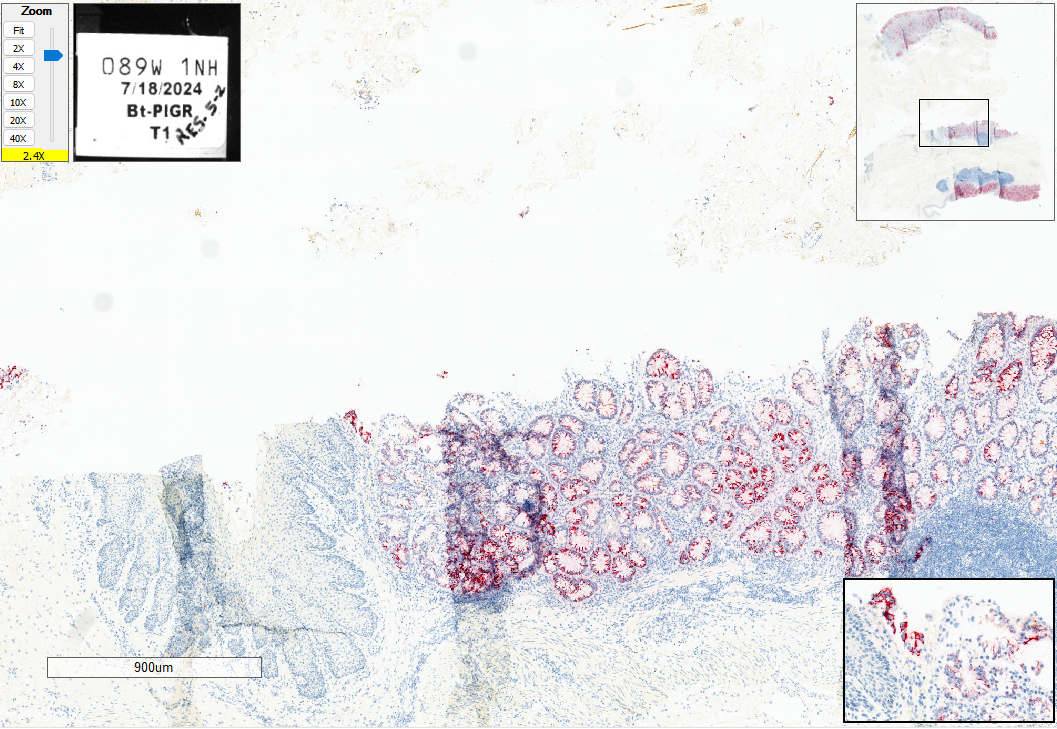

Supplement: S2 Data — (ZIP) [file ppat.1013584.s011.zip › S2_Data/Test/PIGR_5-2.png]

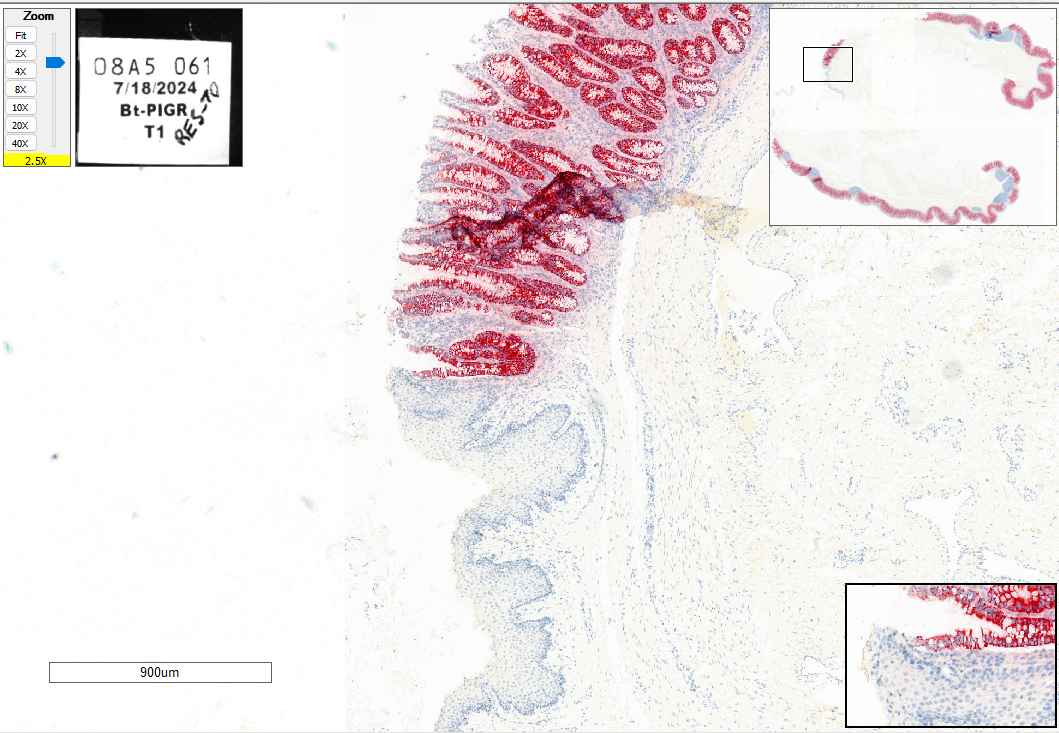

Supplement: S2 Data — (ZIP) [file ppat.1013584.s011.zip › S2_Data/Test/PIGR_T0.png]

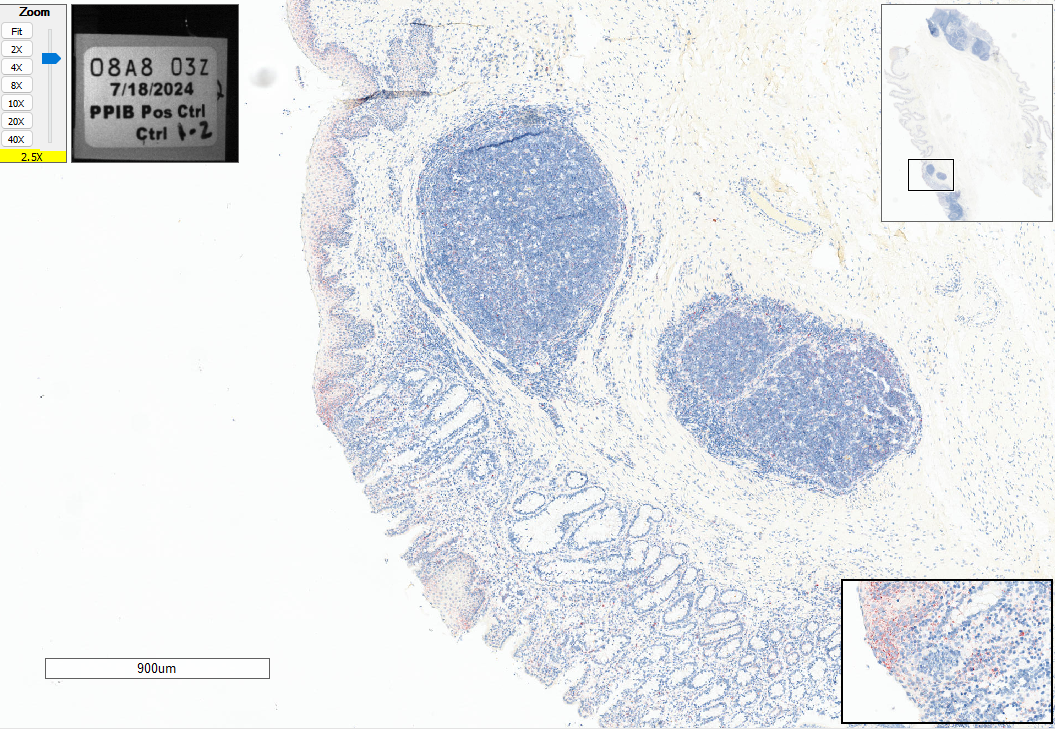

Supplement: S2 Data — (ZIP) [file ppat.1013584.s011.zip › S2_Data/Test/PPIB_1-2.png]

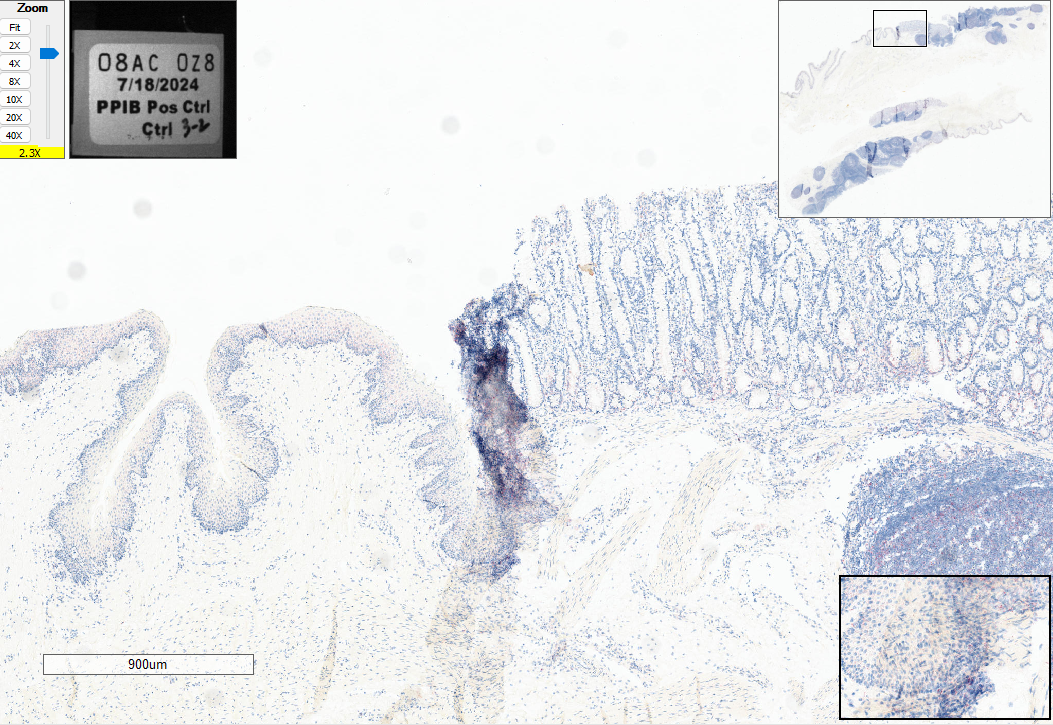

Supplement: S2 Data — (ZIP) [file ppat.1013584.s011.zip › S2_Data/Test/PPIB_3-2.png]

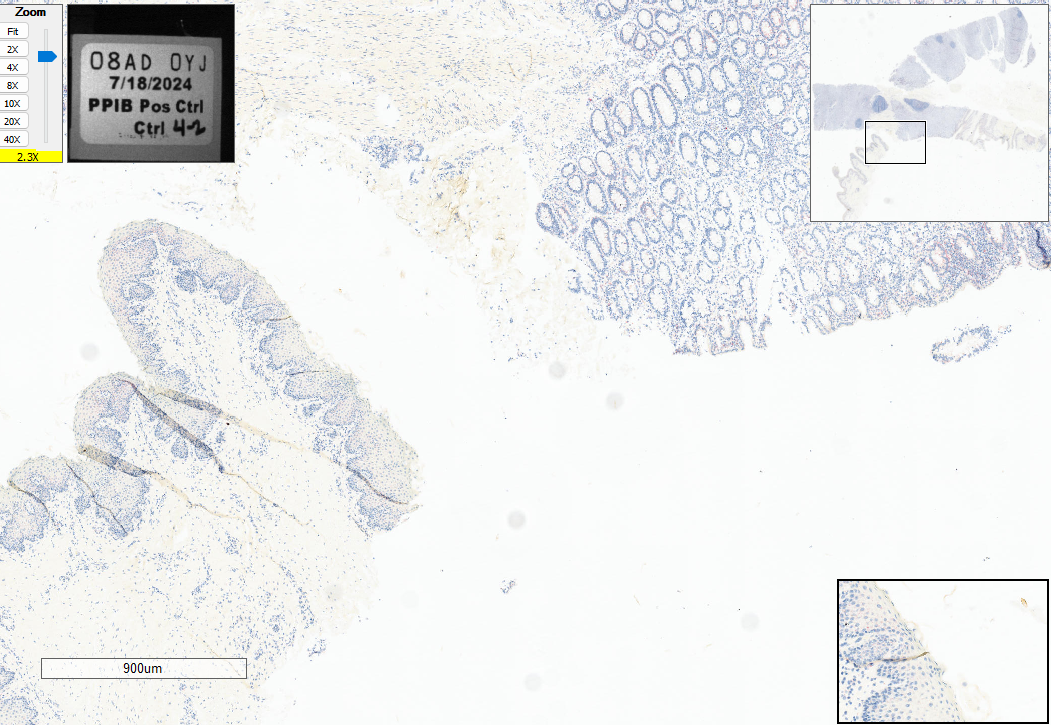

Supplement: S2 Data — (ZIP) [file ppat.1013584.s011.zip › S2_Data/Test/PPIB_4-2.png]

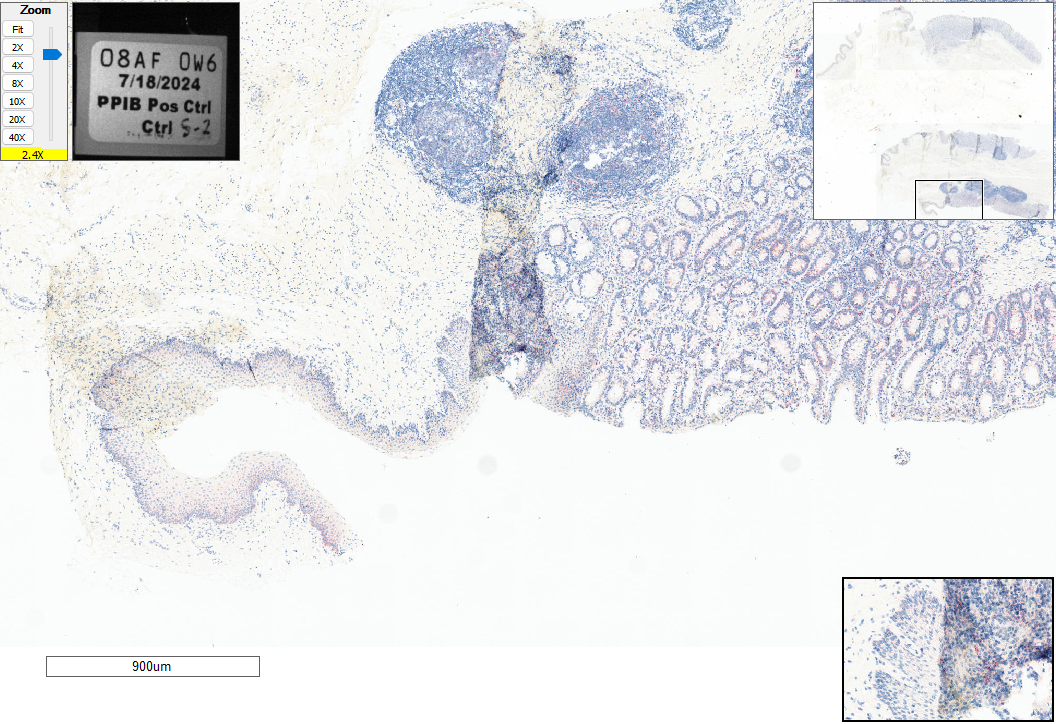

Supplement: S2 Data — (ZIP) [file ppat.1013584.s011.zip › S2_Data/Test/PPIB_5-2.png]

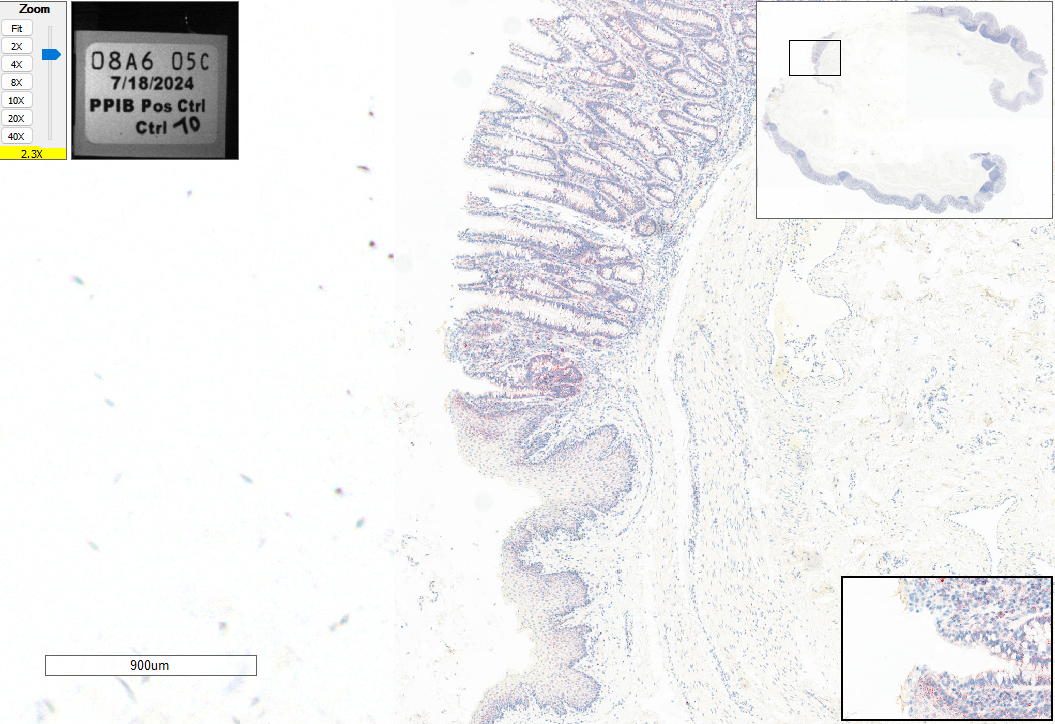

Supplement: S2 Data — (ZIP) [file ppat.1013584.s011.zip › S2_Data/Test/PPIB_T0.png]

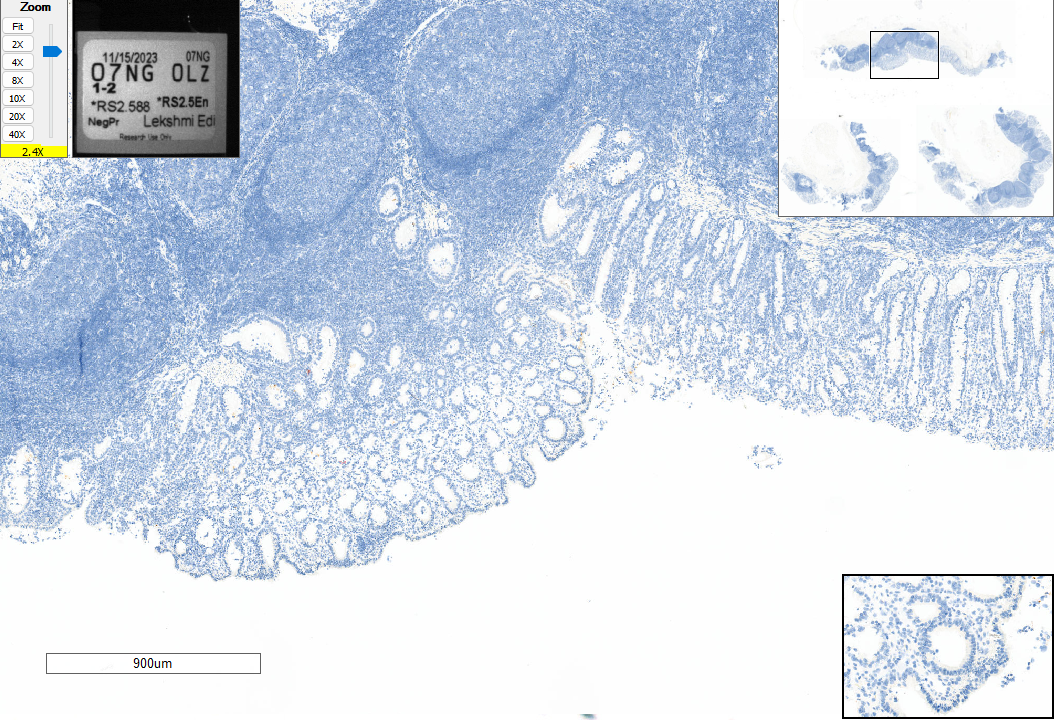

Supplement: S2 Data — (ZIP) [file ppat.1013584.s011.zip › S2_Data/Trial Run/1-2 NegCtrl.png]

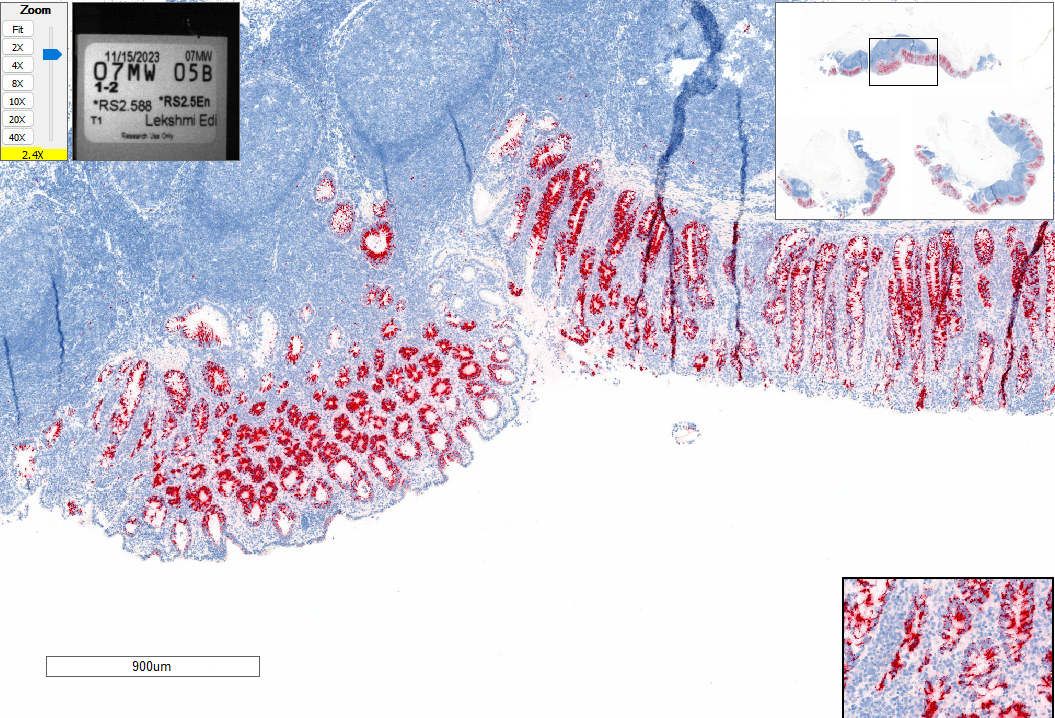

Supplement: S2 Data — (ZIP) [file ppat.1013584.s011.zip › S2_Data/Trial Run/1-2 PIGR.png]

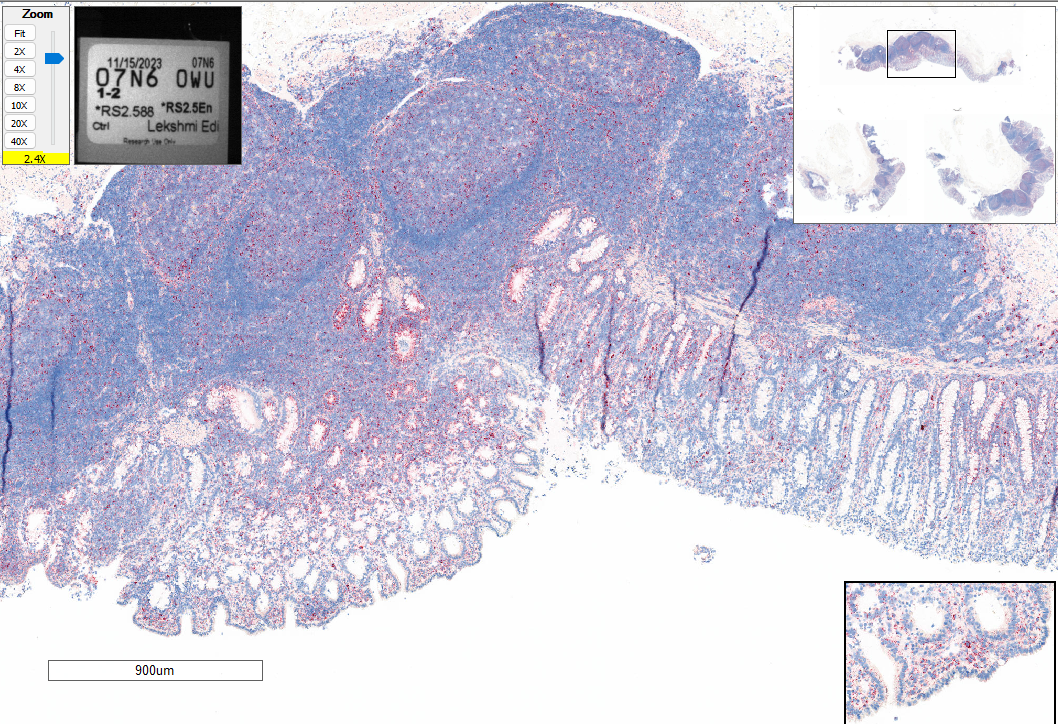

Supplement: S2 Data — (ZIP) [file ppat.1013584.s011.zip › S2_Data/Trial Run/1-2 PosCtrl.png]

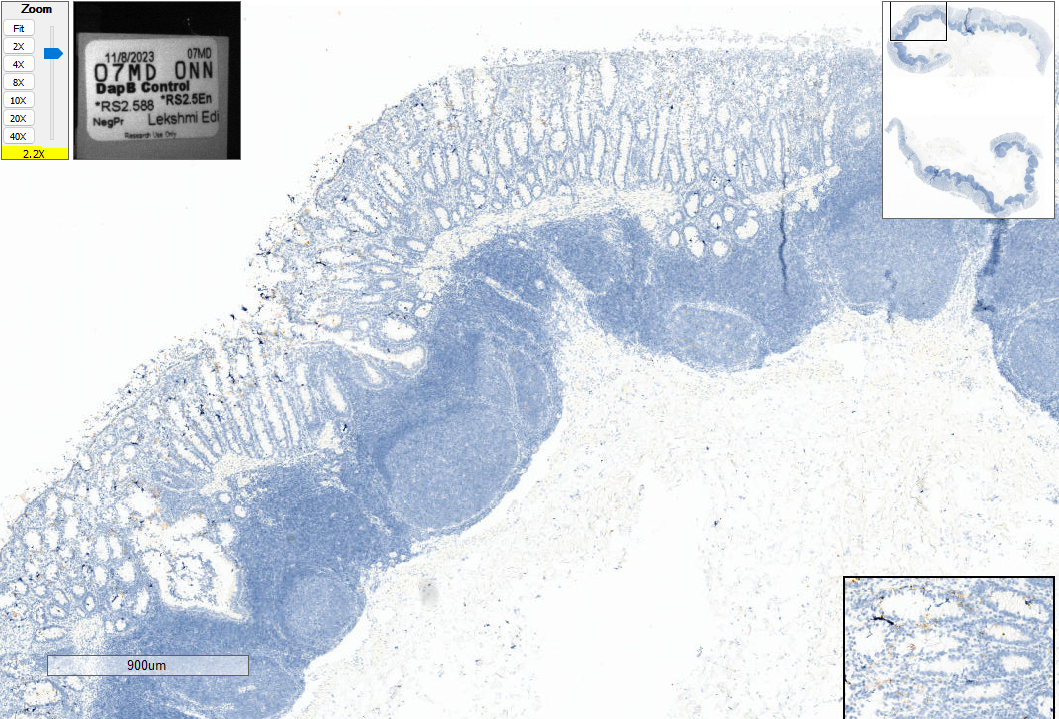

Supplement: S2 Data — (ZIP) [file ppat.1013584.s011.zip › S2_Data/Trial Run/3-2 NegCtrl.png]

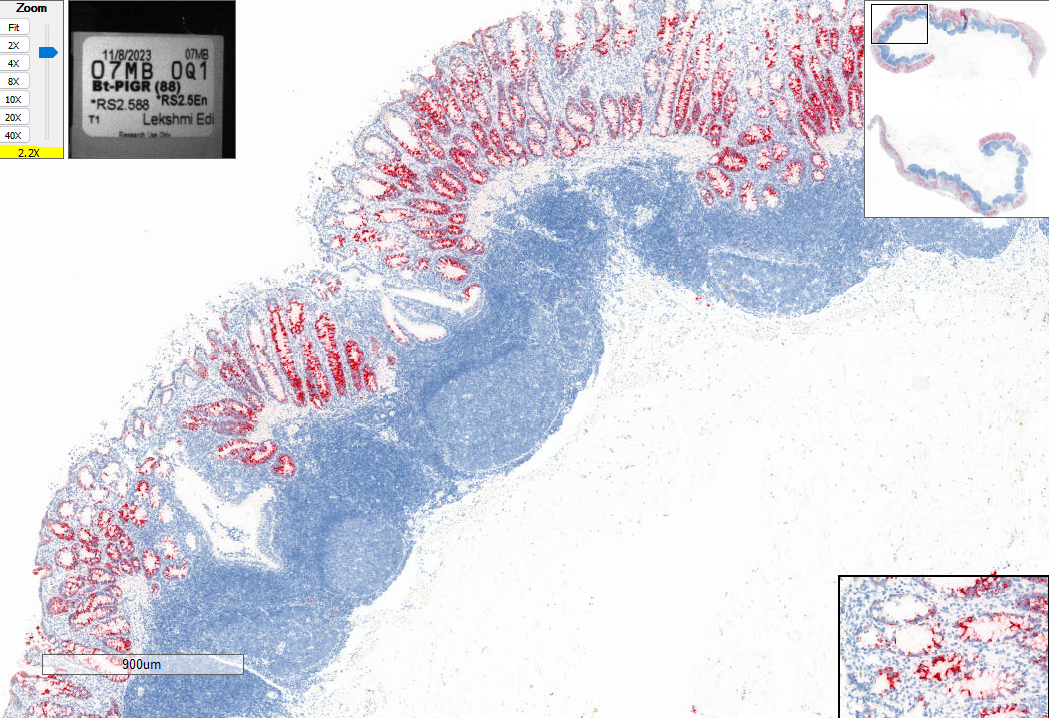

Supplement: S2 Data — (ZIP) [file ppat.1013584.s011.zip › S2_Data/Trial Run/3-2 PIGR.png]

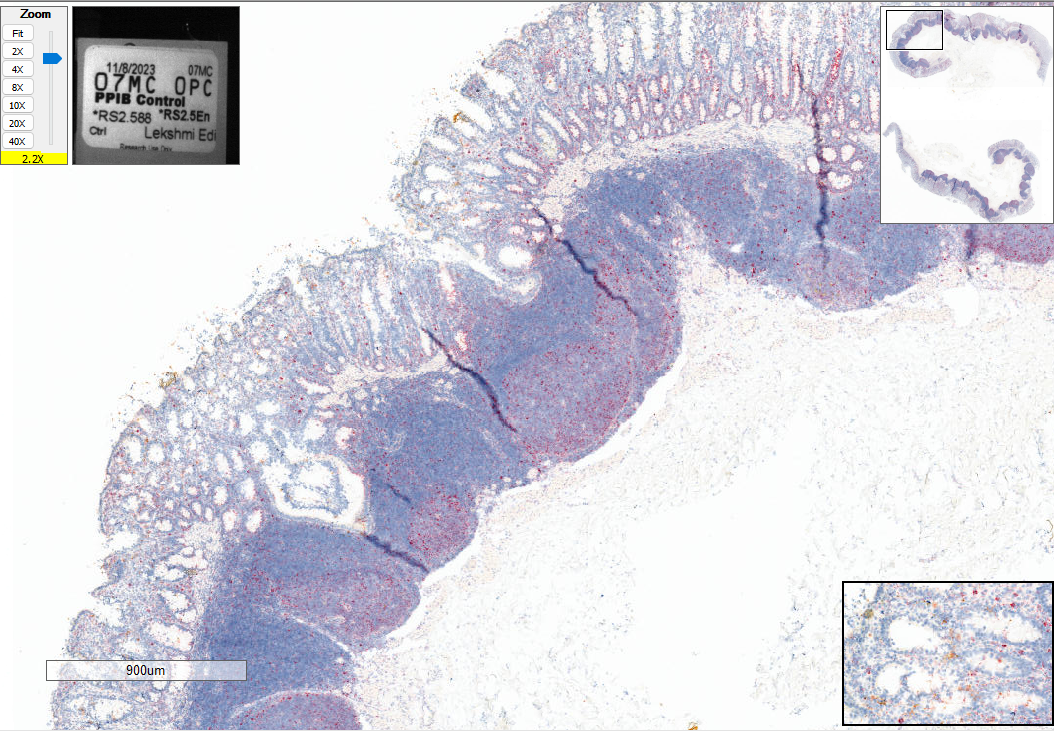

Supplement: S2 Data — (ZIP) [file ppat.1013584.s011.zip › S2_Data/Trial Run/3-2 PosCtrl.png]

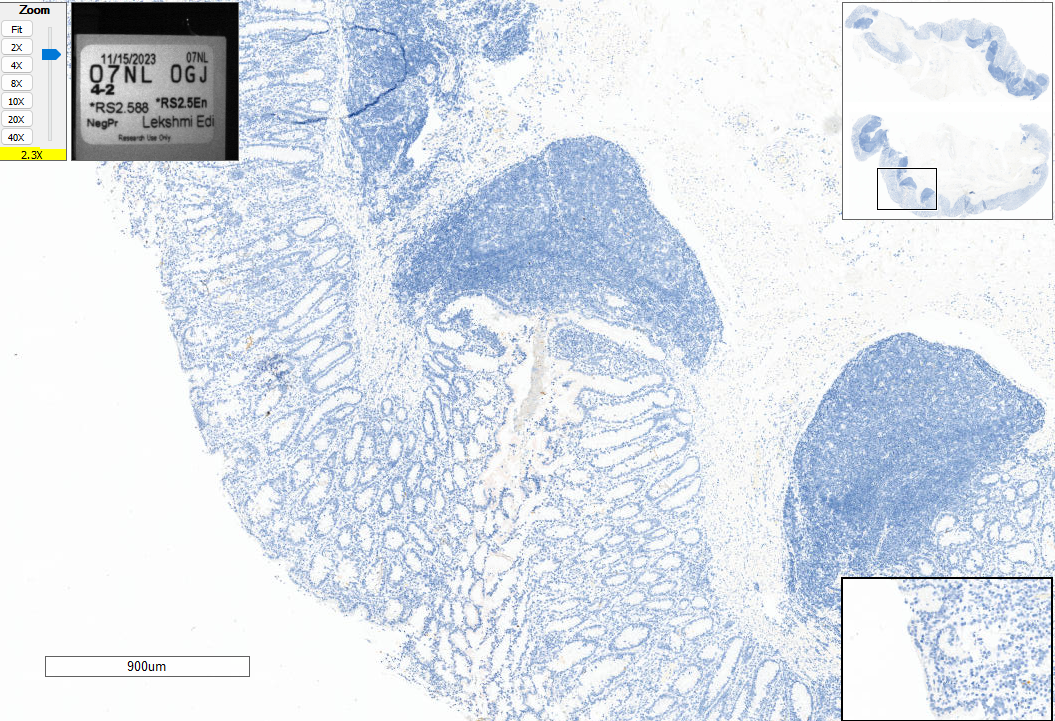

Supplement: S2 Data — (ZIP) [file ppat.1013584.s011.zip › S2_Data/Trial Run/4-2 NegCtrl.png]

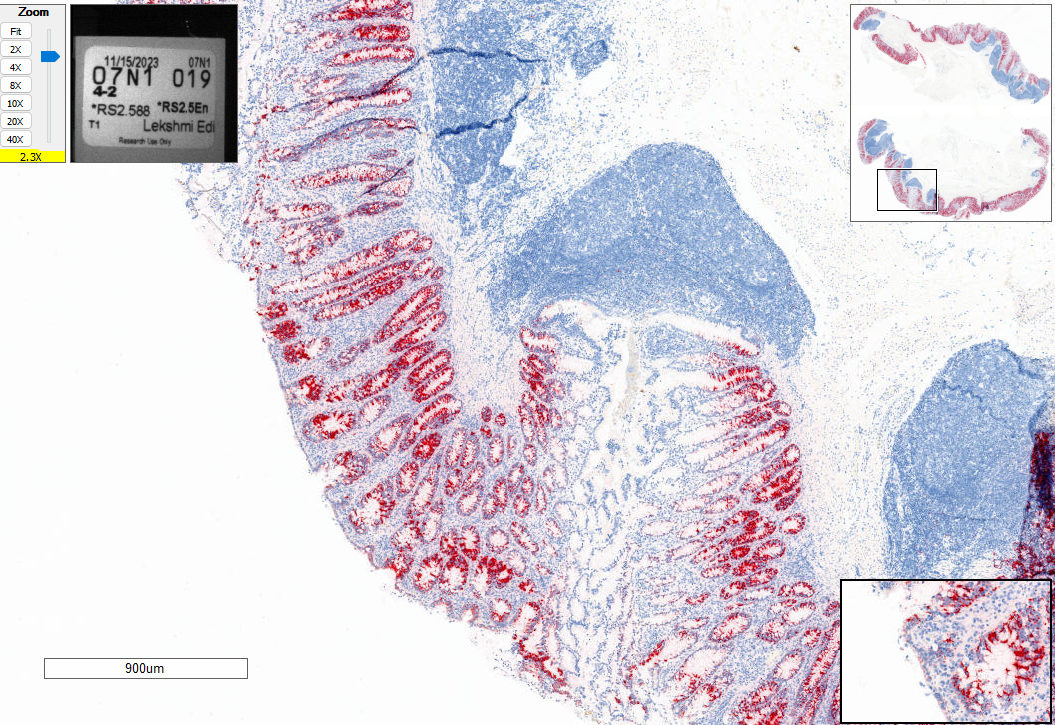

Supplement: S2 Data — (ZIP) [file ppat.1013584.s011.zip › S2_Data/Trial Run/4-2 PIGR.png]

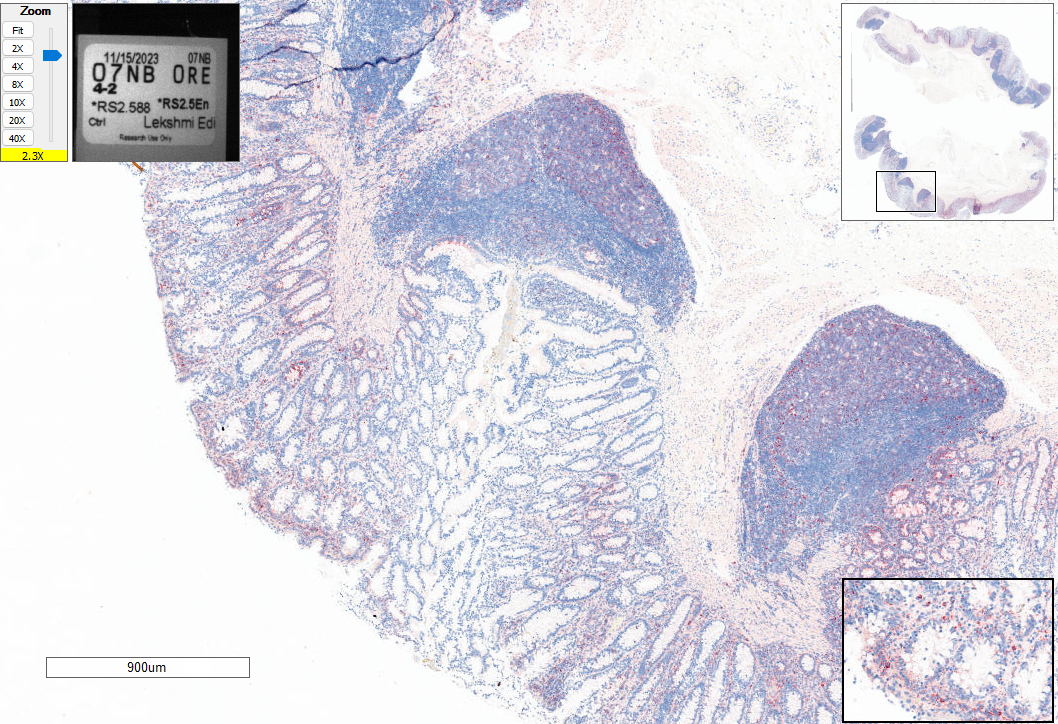

Supplement: S2 Data — (ZIP) [file ppat.1013584.s011.zip › S2_Data/Trial Run/4-2 PosCtrl.png]

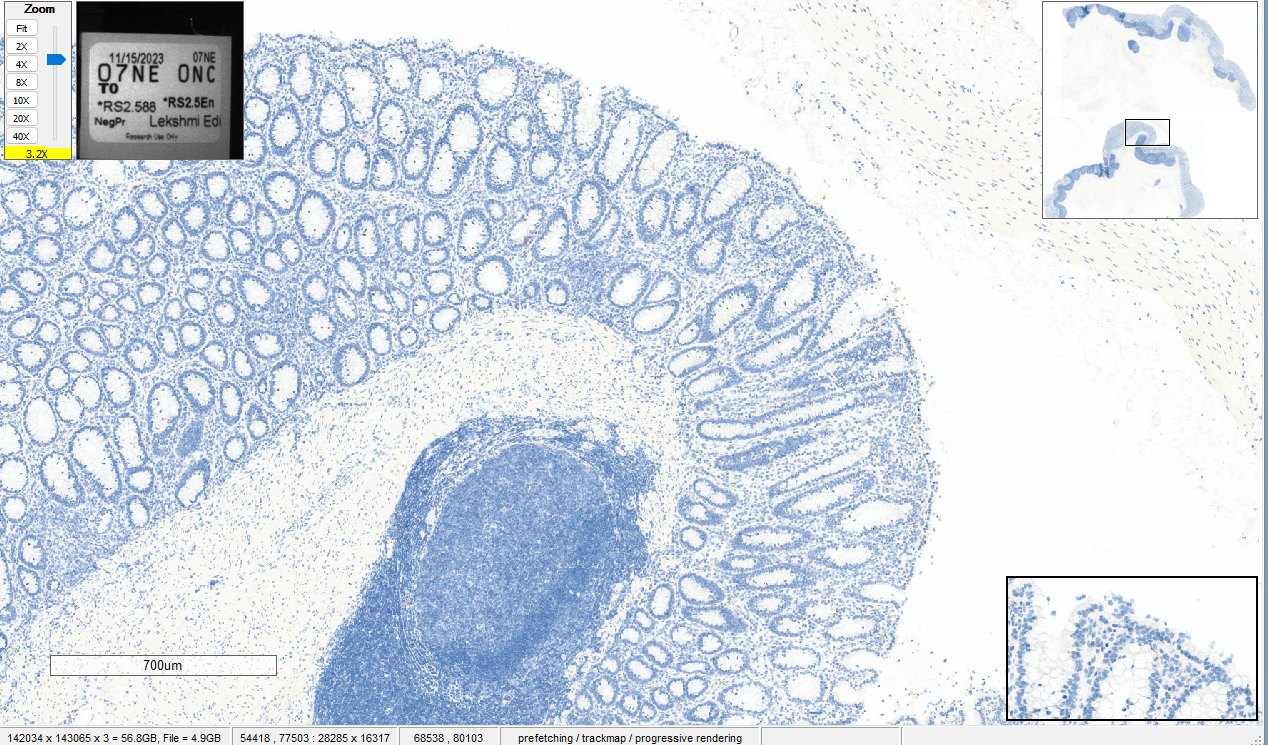

Supplement: S2 Data — (ZIP) [file ppat.1013584.s011.zip › S2_Data/Trial Run/T0 NegCtrl.png]

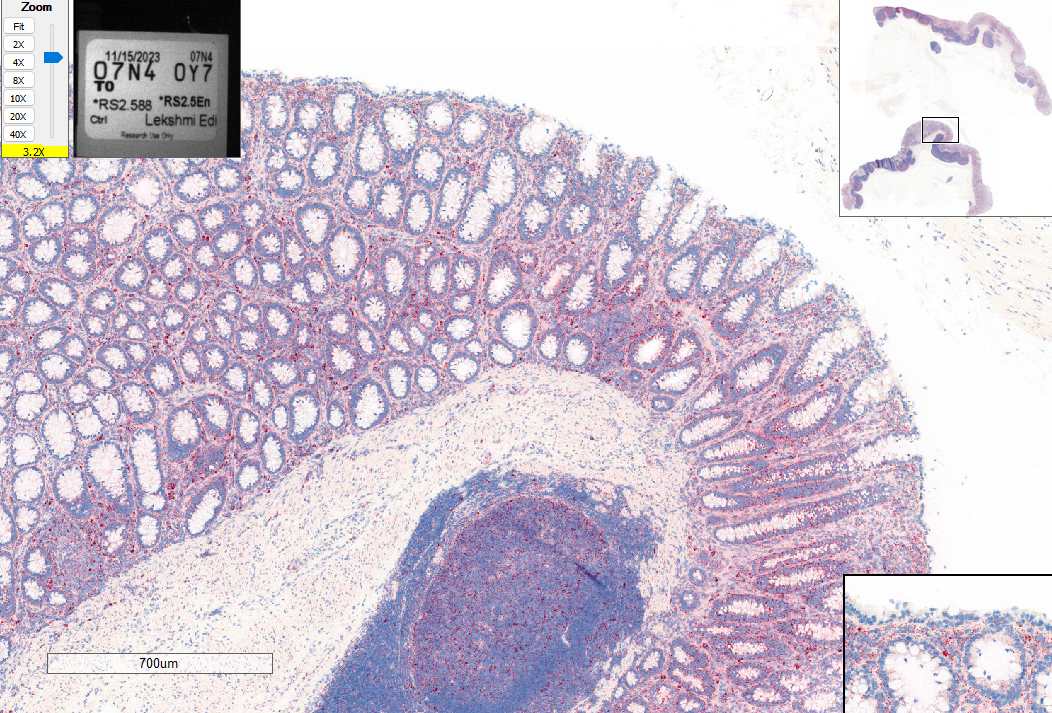

Supplement: S2 Data — (ZIP) [file ppat.1013584.s011.zip › S2_Data/Trial Run/T0 PIGR.png]

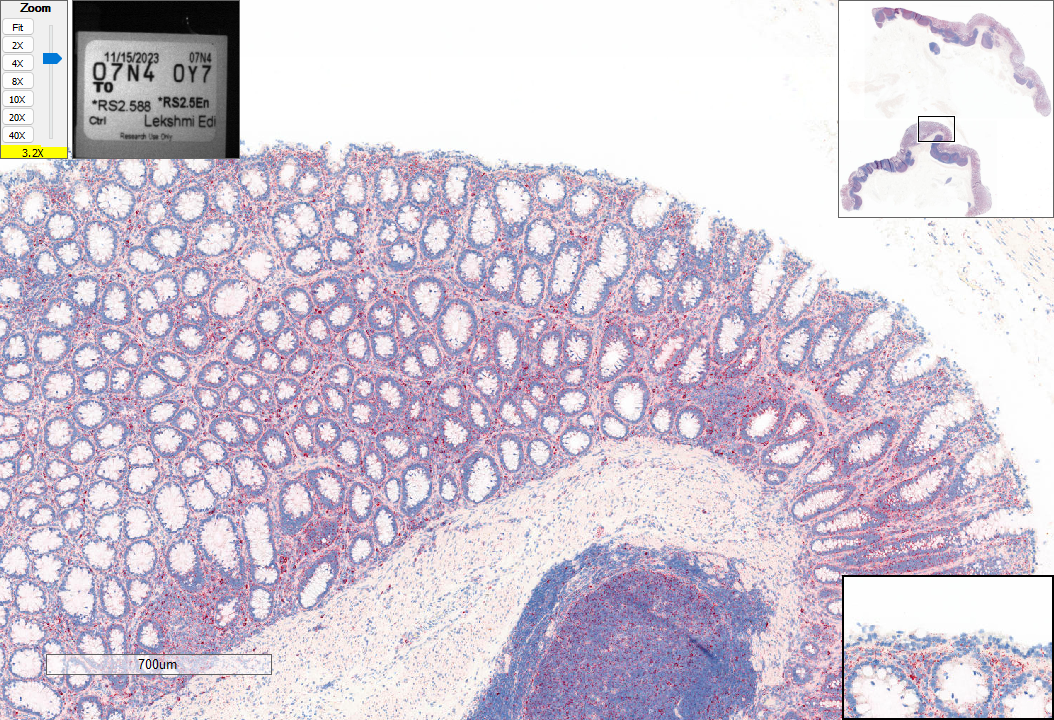

Supplement: S2 Data — (ZIP) [file ppat.1013584.s011.zip › S2_Data/Trial Run/T0 PosCtrl.png]
