# Supplementary material for: The carbon starvation-inducible lipoprotein (Slp) influences differential adherence of Escherichia coli O157:H7 at the bovine rectoanal junction
Source: PLoS Pathog. 2026 May 18;22(5):e1013584. doi: 10.1371/journal.ppat.1013584 (PMC13193606; doi:10.1371/journal.ppat.1013584)
Supplement: S2 Table — (DOCX) [file ppat.1013584.s008.docx]

**S2A.** Representative PATS profiles of isolates inoculated and recovered from RAJ-IVOCs.

| **Strain** | **Polymorphic *Xba*I sites** | | | | | | | | **Polymorphic *Avr*II sites** | | | | | | | **Virulence Genes** | | | |
| --- | --- | --- | --- | --- | --- | --- | --- | --- | --- | --- | --- | --- | --- | --- | --- | --- | --- | --- | --- |
|  | **IK8** | **IK25** | **IK114** | **IK118** | **IK123** | **IK127** | **IKB3** | **IKB5** | **IKNR3** | **IKNR7** | **IKNR10** | **IKNR12** | **IKNR16** | **IKNR27** | **IKNR33** | ***stx1*** | ***stx2*** | ***eaeA*** | ***hlyA*** |
| EDL932-WT | 0 | 0 | 1 | 1 | 1 | 1 | 1 | 1 | 2 | 2 | 2 | 2 | 2 | 2 | 2 | 1 | 1 | 1 | 1 |
| EDL932 Δ*slp* | 0 | 0 | 1 | 1 | 1 | 1 | 1 | 1 | 2 | 2 | 2 | 2 | 2 | 2 | 2 | 1 | 1 | 1 | 1 |
| EDL932 Δ*slp*-p:*slp* | 0 | 0 | 1 | 1 | 1 | 1 | 1 | 1 | 2 | 2 | 2 | 2 | 2 | 2 | 2 | 1 | 1 | 1 | 1 |

**S2B.** Representative PCR profiles of isolates recovered from RAJ-IVOCs: Post assay, in leftover inoculum (post) and from non-enrichment bacterial culture of tissues (TC-NE).

| **Isolate** | ***slp*** | **pUC18** | ***stx2*** | **Match with the corresponding test strain** |
| --- | --- | --- | --- | --- |
| EDL932-WT-A post | + | - | + | ✓ |
| EDL932-WT-B post | + | - | + | ✓ |
| EDL932 Δ*slp*-A post | - | - | + | ✓ |
| EDL932 Δ*slp*-B post | - | - | + | ✓ |
| EDL932 Δ*slp*-p:*slp*-A post | + | + | + | ✓ |
| EDL932 Δ*slp*-p:*slp*-B post | + | + | + | ✓ |
| EDL932-WT-A TC-NE | + | - | + | ✓ |
| EDL932-WT-B TC-NE | + | - | + | ✓ |
| EDL932 Δ*slp*-A TC-NE | - | - | + | ✓ |
| EDL932 Δ*slp*-B TC-SE | - | - | + | ✓ |
| EDL932Δ*slp*-p:*slp*-A TC-SE | + | + | + | ✓ |
| EDL932Δ*slp*-p:*slp*-B TC-NE | + | + | + | ✓ |
