# Supplementary material for: The carbon starvation-inducible lipoprotein (Slp) influences differential adherence of Escherichia coli O157:H7 at the bovine rectoanal junction
Source: PLoS Pathog. 2026 May 18;22(5):e1013584. doi: 10.1371/journal.ppat.1013584 (PMC13193606; doi:10.1371/journal.ppat.1013584)
Supplement: S1 Data — (ZIP) [file ppat.1013584.s010.zip › S1_Data/CassmannResults_IVOC_011123-012423-030123.pdf]

Project ID# 703: Kudva IVOC 011123
PRE-ASSAY

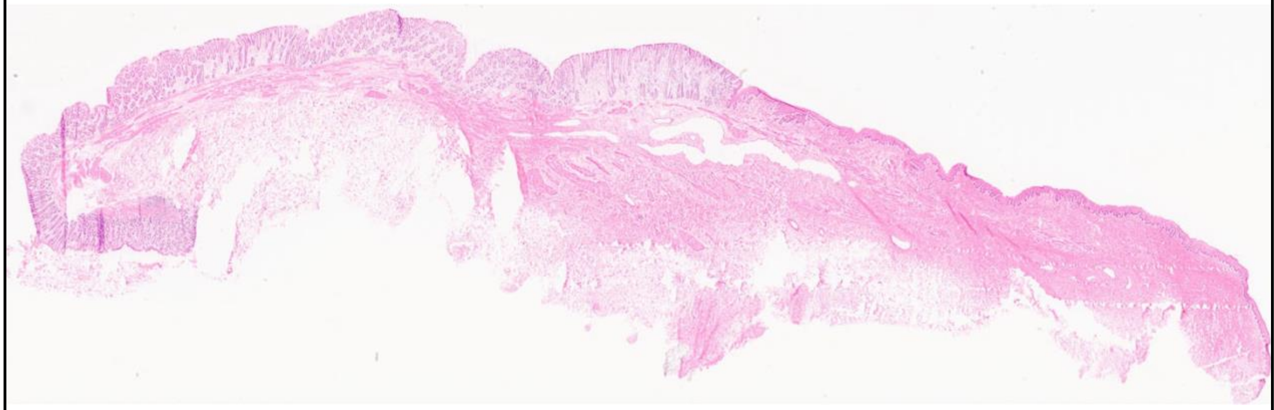

H&E-stained frozen section of recto-anal junction that contains squamous epithelium and mucosal columnar epithelial cells with abundant goblet cells. The mucosal portion of tissue contains mucosa, submucosa, circular smooth muscle of the tunic muscularis, and tunica adventitia

Within the submucosa, there is a single partial edge of a lymphoid follicle at the oral end of the tissue.

Cellular staining is good.

The superficial mucosal epithelium is completely intact. There is mildly folding artifact. Mucosal integrity is good. In the squamous epithelium there is mild intercellular bridging (intercellular edema) and intracellular vacuolation of squamous epithelial cells (intracellular edema).

Project ID# 703: Kudva IVOC 011123
PRE-ASSAY

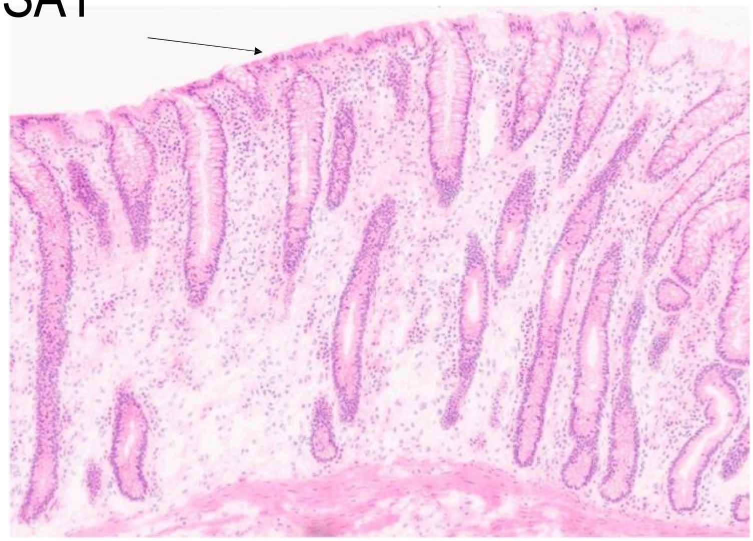

Good surface columnar epithelium (arrow).
Epithelium lining is intact throughout entire length of crypt.

Project ID# 703: Kudva IVOC 011123 NB

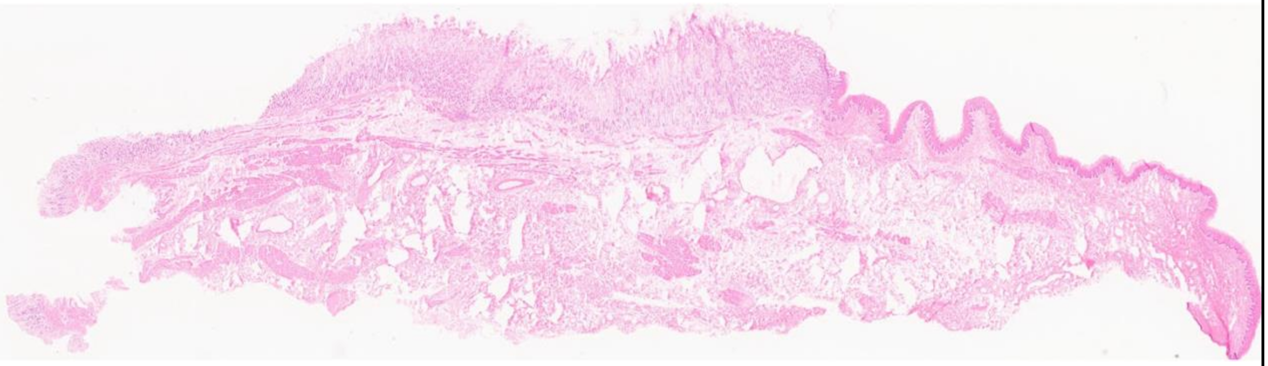

H&E-stained frozen section of recto-anal junction that contains squamous epithelium and mucosal columnar epithelial cells with abundant goblet cells. The mucosal portion of tissue contains mucosa, submucosa, circular smooth muscle of the tunic muscularis, and tunica adventitia

Within the submucosa, there is a single partial edge of a lymphoid follicle.

Cellular staining is good.

The superficial mucosal epithelium is mostly absent. Full thickness mucosa is focally eroded and there is disruption in the superficial ¼ of the mucosa elsewhere. In the squamous epithelium there is mild intercellular bridging (intercellular edema) and intracellular vacuolation of squamous epithelial cells (intracellular edema).

There is mild inflammatory infiltrate in the mucosal lamina propria consisting of lymphocytes and plasma cells. Some deep crypts contain polymorphonuclear cells (likely neutrophils).

Project ID# 703: Kudva IVOC 011123 ED L932

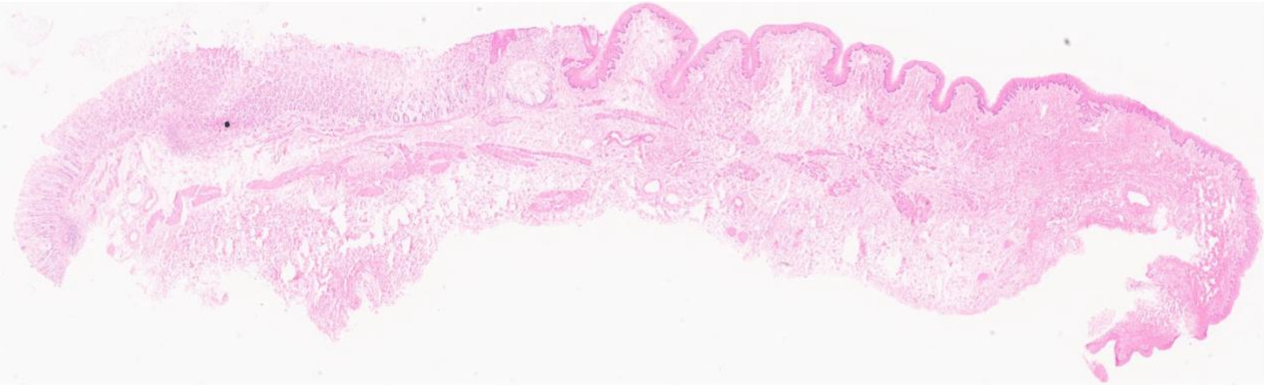

H&E-stained frozen section of recto-anal junction that contains squamous epithelium and mucosal columnar epithelial cells with abundant goblet cells. The section is slightly tangentially oriented leading to cross-sectioning of mucosal crypts. The mucosal portion of tissue contains mucosa, submucosa, circular smooth muscle of the tunic muscularis, and tunica adventitia

Within the submucosa, there are two partial edges of a lymphoid follicles.

Cellular staining is good.

The superficial mucosal epithelium is mostly absent except for 2 foci with surface epithelium present. Other than loss of surface columnar epithelium, the mucosa and lamina propria is mostly present and intact. In the squamous epithelium there is mild intercellular bridging (intercellular edema) and intracellular vacuolation of squamous epithelial cells (intracellular edema).

There is mild inflammatory infiltrate in the mucosal lamina propria consisting of

lymphocytes and plasma cells. The superficial dermis has a slight increase in numbers of macrophages and lymphocytes underlying the squamous epithelium.

Project ID# 703: Kudva IVOC 011123
EDL 932 ∆SLP

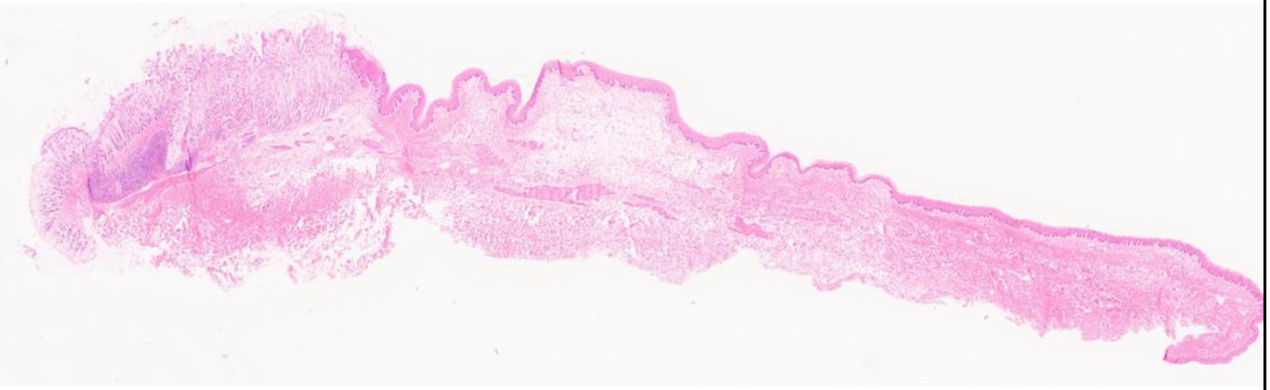

H&E-stained frozen section of recto-anal junction that contains squamous epithelium and mucosal columnar epithelial cells with abundant goblet cells. The mucosal portion comprises roughly 20% of the tissue. The mucosal portion of tissue contains mucosa, submucosa, circular smooth muscle of the tunic muscularis, and tunica adventitia

Within the submucosa, there is a large lymphoid nodule.

Cellular staining is good.

The superficial mucosal epithelium is intact in ~20% of the mucosa. Other than loss of surface columnar epithelium, the mucosa and lamina propria is mostly present and intact. In the squamous epithelium there is mild intercellular bridging (intercellular edema) and intracellular vacuolation of squamous epithelial cells (intracellular edema).

There is mild inflammatory infiltrate in the mucosal lamina propria consisting of

lymphocytes and plasma cells. Occasionally there are round cells with discrete eosinophilic globules resembling Russell Bodies (Ig inclusions). These cells are consistent with Mott Cells.

Project ID# 703: Kudva IVOC 011123
EDL 932 ΔSLP

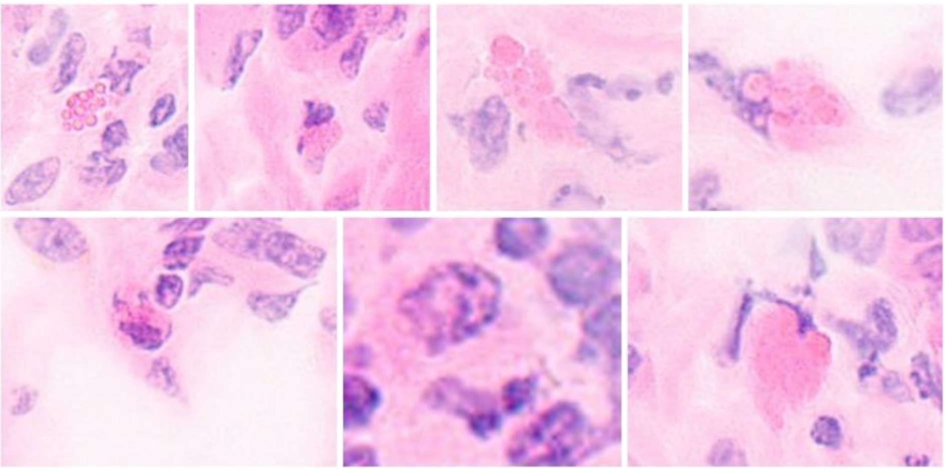

Occasionally there are round cells with discrete eosinophilic globules resembling Russell Bodies (Ig inclusions). These cells are consistent with Mott Cells.

Project ID# 703: Kudva IVOC 011123
EDL 932 COMP

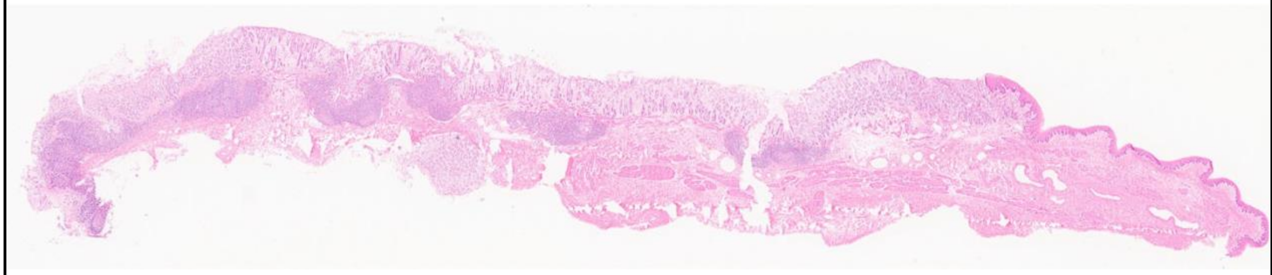

H&E-stained frozen section of recto-anal junction that contains squamous epithelium and mucosal columnar epithelial cells with abundant goblet cells. The mucosal portion of tissue contains mucosa, submucosa, circular smooth muscle of the tunic muscularis, and tunica adventitia. There is folding of the tissue – mucosal portion visible below the muscle layers focally.

Within the submucosa, there is abundant lymphoid tissue with follicles formed. Lymphoid tissue is hyperplastic and spans ~80% of mucosa.

Cellular staining is good.

The superficial mucosal epithelium is intact in ~40-50% of the mucosa. Other than loss of surface columnar epithelium, the mucosa and lamina propria is mostly present and intact. There is loss/separation of cyrpts multifocally. They are separated by white space (edema) fibrous connective tissue and inflammatory cells – macrophages, lymphocytes, plasma cells and occasional mott cells.

This animal had antemortem granulomatous lymphoplasmacytic proctitis/colitis.

In the squamous epithelium there is mild intercellular bridging (intercellular edema) and intracellular vacuolation of squamous epithelial cells (intracellular edema).

Project ID# 703: Kudva IVOC 011123
EDL 932 COMP

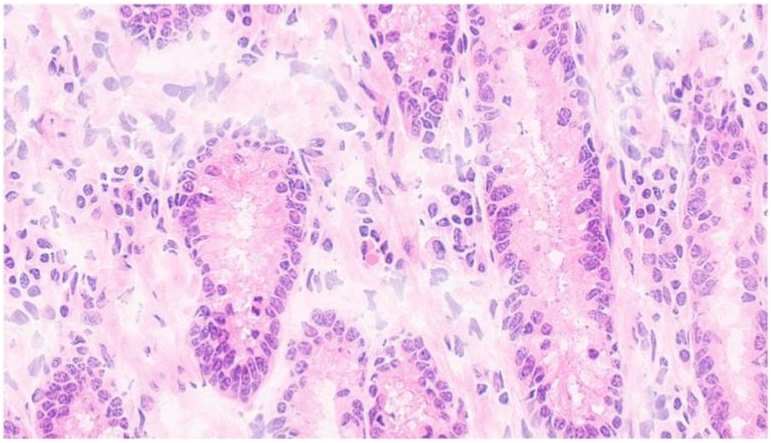

Increased lymphocytes, plasma cells, and macrophages in the mucosal lamina propria

Project ID# 704: Kudva IVOC 012623
PRE-ASSAY

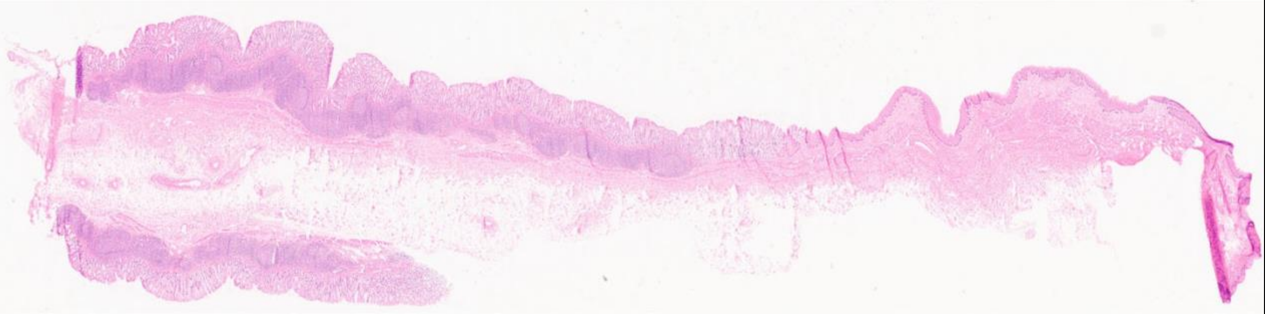

H&E-stained frozen section of recto-anal junction that contains squamous epithelium and mucosal columnar epithelial cells with abundant goblet cells. The mucosal portion of tissue contains mucosa, submucosa, circular smooth muscle of the tunic muscularis, and tunica adventitia. There is a focal folding artifact as the tissue curves.

Within the submucosa, there is abundant lymphoid tissue with follicles formed. Lymphoid tissue is hyperplastic and spans >90% of mucosa.

Cellular staining is good.

The superficial mucosal epithelium is intact. Other than loss of surface columnar epithelium, the mucosa and lamina propria is present and intact. There is expansion of lamina propria. Crypts are separated by white space (edema) and a large infiltrate of inflammatory cells – macrophages, epithelioid macrophages, lymphocytes, plasma cells and mott cells. There are rare multinucleated macrophages. There are crypt abscesses.

This animal had antemortem granulomatous lymphoplasmacytic, neutrophilic proctitis/colitis and lymphoid hyperplasia. The presence of epithelioid macrophages and multinucleated cells is consistent with Johne’s disease. PCR of tissue may help confirm the presence of Mycobacterium avium spp paratuberculosis.

In the squamous epithelium there is mild intercellular bridging (intercellular edema) and intracellular vacuolation of squamous epithelial cells (intracellular edema).

Project ID# 704: Kudva IVOC 012623 PRE-ASSAY

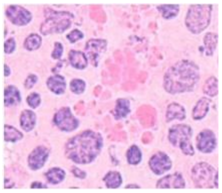

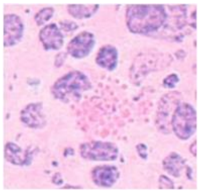

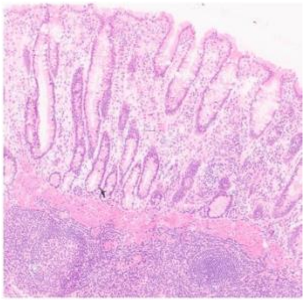

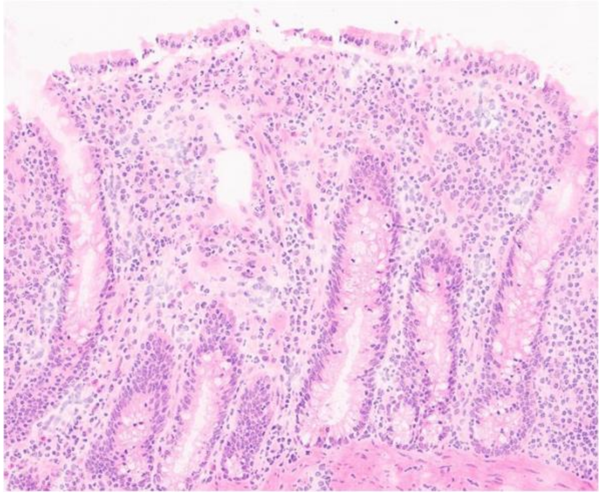

This animal had antemortem granulomatous lymphoplasmacytic proctitis/colitis and lymphoid hyperplasia. The presence of epithelioid macrophages and multinucleated cells is consistent with Johne’s disease. PCR of tissue may help confirm the presence of Mycobacterium avium spp paratuberculosis.

Project ID# 704: Kudva IVOC 012623 NB

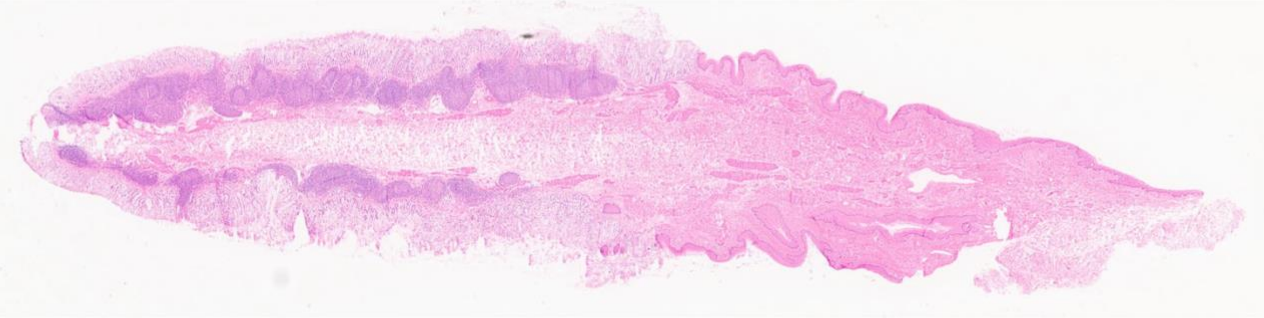

H&E-stained frozen section of recto-anal junction that contains squamous epithelium and mucosal columnar epithelial cells with abundant goblet cells. The mucosal portion of tissue contains mucosa, submucosa, circular smooth muscle of the tunic muscularis, and tunica adventitia. The embedded tissue is curved such that mucosa is seen on the top and bottom.

Within the submucosa, there is abundant lymphoid tissue with follicles formed. Lymphoid tissue is hyperplastic and spans >90% of mucosa.

Cellular staining is good.

The superficial mucosal epithelium is mostly missing. Other than loss of surface columnar epithelium, the mucosa and lamina propria is present and intact. There is expansion of lamina propria. Crypts are separated by white space (edema) and a large infiltrate of inflammatory cells – macrophages, lymphocytes, plasma cells and mott cells and rare neutrophils. There are many Mott Cells within germinal centers of lymphoid follicles. The findings suggest a chronic inflammatory stimulus.

This animal had antemortem granulomatous lymphoplasmacytic proctitis/colitis and lymphoid hyperplasia with numerous Mott Cells present.

In the squamous epithelium there is mild intercellular bridging (intercellular edema) and intracellular vacuolation of squamous epithelial cells (intracellular edema).

Project ID# 704: Kudva IVOC 012623 NB

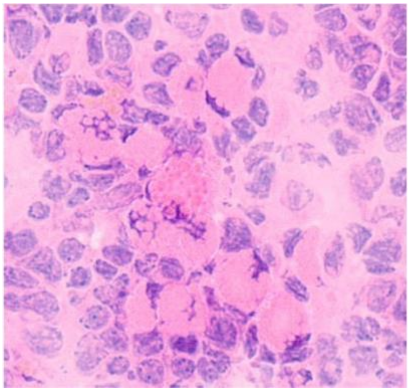

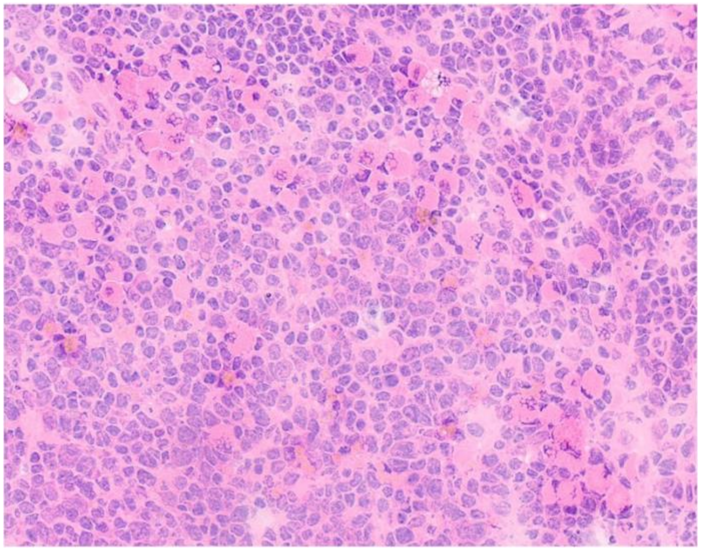

There are many Mott Cells within germinal centers of lymphoid follicles. Others are within lamina propria/mucosa. The findings suggest a chronic inflammatory stimulus.

Project ID# 704: Kudva IVOC 012623 NB

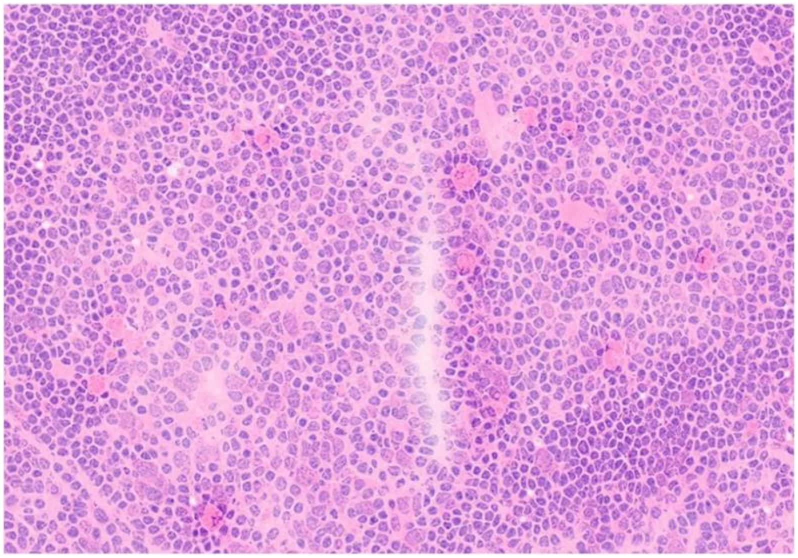

There are many Mott Cells within germinal centers of lymphoid follicles. Others are within lamina propria/mucosa. The findings suggest a chronic inflammatory stimulus.

Project ID# 704: Kudva IVOC 012623
EDL 932

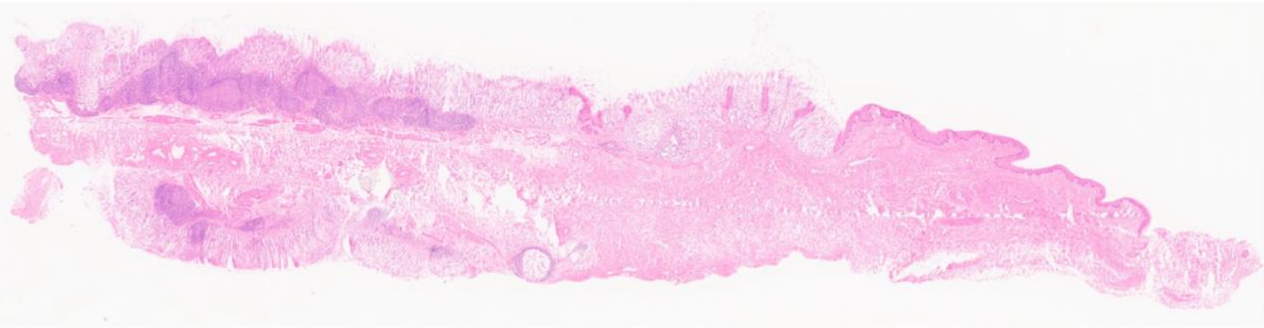

H&E-stained frozen section of recto-anal junction that contains squamous epithelium and mucosal columnar epithelial cells with abundant goblet cells. The mucosal portion of tissue contains mucosa, submucosa, circular smooth muscle of the tunic muscularis, and tunica adventitia. The embedded tissue is curved such that mucosa is seen on the top and bottom, and at the RAJ there is a mix of squamous epithelium and mucosa.

Within the submucosa, there is abundant lymphoid tissue with follicles formed. Lymphoid tissue is hyperplastic and spans >60% of mucosa.

Cellular staining is good.

The superficial mucosal epithelium is mostly missing. Other than loss of surface columnar epithelium, the mucosa and lamina propria is present and intact in the bottom 2/3 of mucosa. There is expansion of lamina propria. Crypts are separated by white space (edema) and a large infiltrate of inflammatory cells – lymphocytes, plasma cells, neutrophils and macrophages. The polymorphonuclear cells seen are

likely a mix of eosinophils and neutrophils. Some large granular lymphocytes are observed.

This animal had antemortem lymphoplasmacytic proctitis/colitis and lymphoid hyperplasia.

In the squamous epithelium there is mild intercellular bridging (intercellular edema) and intracellular vacuolation of squamous epithelial cells (intracellular edema).

Project ID# 704: Kudva IVOC 012623
EDL 932 ΔSLP

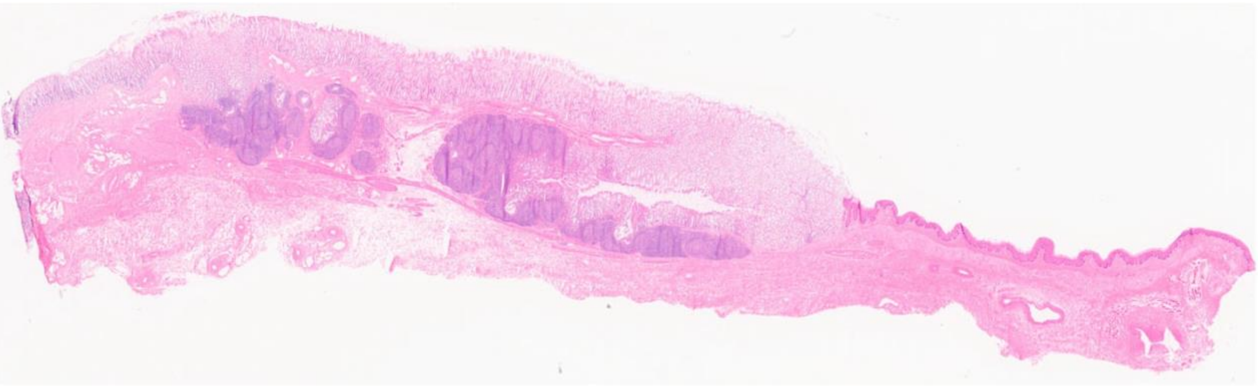

H&E-stained frozen section of recto-anal junction that contains squamous epithelium and mucosal columnar epithelial cells with abundant goblet cells. The mucosal portion of tissue contains mucosa, submucosa, circular smooth muscle of the tunic muscularis, and tunica adventitia.

Within the submucosa, there is abundant lymphoid tissue with follicles formed. Lymphoid tissue is hyperplastic and spans >90% of mucosa.

Cellular staining is good.

The superficial mucosal epithelium is missing. Other than loss of surface columnar epithelium, the mucosa and lamina propria is present and intact in the bottom 50% of mucosa. There is expansion of lamina propria. Crypts are separated by a large infiltrate of inflammatory cells – lymphocytes, plasma cells, neutrophils and macrophages – There are rare small multinucleated macrophages. The polymorphonuclear cells seen are likely a mix of eosinophils and neutrophils. Some large granular lymphocytes are observed.

This animal had antemortem lymphoplasmacytic granulomatous proctitis/colitis and lymphoid hyperplasia.

In the squamous epithelium there is mild intercellular bridging (intercellular edema) and intracellular vacuolation of squamous epithelial cells (intracellular edema).

Project ID# 704: Kudva IVOC 012623
EDL 932 COMP

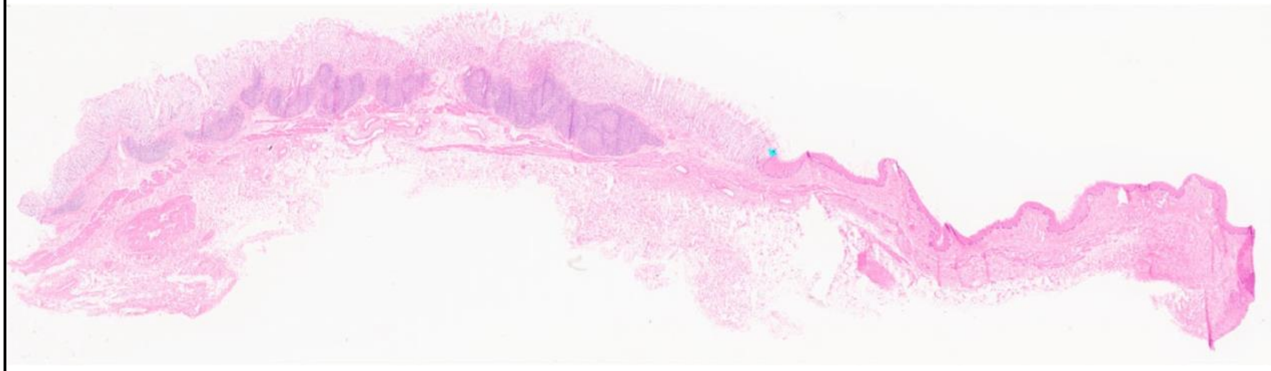

H&E-stained frozen section of recto-anal junction that contains squamous epithelium and mucosal columnar epithelial cells with abundant goblet cells. The mucosal portion of tissue contains mucosa, submucosa, circular smooth muscle of the tunic muscularis, and tunica adventitia. The embedded tissue is curved such that 2 layers of mucosa are seen at the RAJ.

Within the submucosa, there is abundant lymphoid tissue with follicles formed. Lymphoid tissue is hyperplastic and spans >80% of mucosa.

Cellular staining is good.

The superficial mucosal epithelium is mostly missing except for a focal area about 1mm in length. Other than loss of surface columnar epithelium, the mucosa and lamina propria is present and intact in the bottom 90% of mucosa. There is expansion of lamina propria. Crypts are separated by a large infiltrate of inflammatory cells – lymphocytes, plasma cells, neutrophils and macrophages –

some epithelioid macrophages. The polymorphonuclear cells seen are likely a mix of eosinophils and neutrophils. Some large granular lymphocytes are observed.

This animal had antemortem lymphoplasmacytic granulomatous proctitis/colitis and lymphoid hyperplasia.

In the squamous epithelium there is mild intercellular bridging (intercellular edema) and intracellular vacuolation of squamous epithelial cells (intracellular edema).

Project ID# 707: Kudva IVOC 030123
PRE-ASSAY

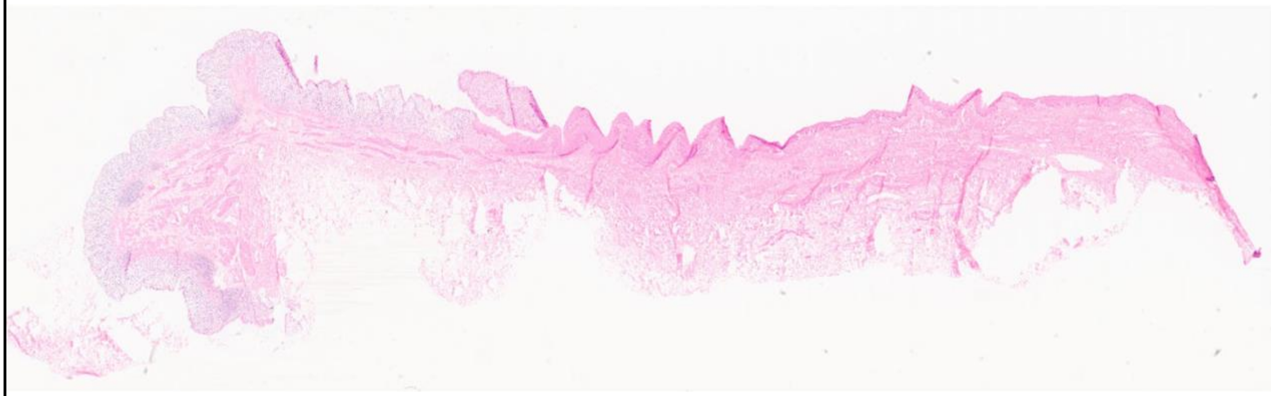

H&E-stained frozen section of recto-anal junction that contains squamous epithelium and mucosal columnar epithelial cells with abundant goblet cells. The mucosal portion of tissue contains mucosa, submucosa, circular smooth muscle of the tunic muscularis, and tunica adventitia.

Within the mucosa and submucosa, there are a few lymphoid nodules – recto-anal mucosal lymphoid tissue (RAMALT).

Cellular staining is good.

The superficial mucosal epithelium is mostly intact; there is rare loss of surface columnar epithelium. Integrity is good; however, there is some mild folding of the mucosal surface and squamous epithelium. In the squamous epithelium there is mild intercellular bridging (intercellular edema) and intracellular vacuolation of squamous epithelial cells (intracellular edema).

There is a minimal infiltrate of inflammatory cells (lymphocytes and plasma cells) in the

deep mucosa and lamina propria – likely native/ resident population.,

Project ID# 707: Kudva IVOC 030123 NB-A

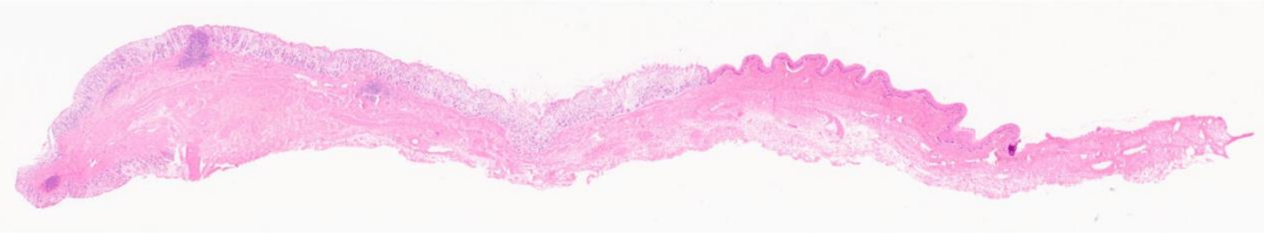

H&E-stained frozen section of recto-anal junction that contains squamous epithelium and mucosal columnar epithelial cells with abundant goblet cells. The mucosal portion of tissue contains mucosa, submucosa, circular smooth muscle of the tunic muscularis, and tunica adventitia.

Within the mucosa and submucosa, there are a few lymphoid nodules – recto-anal mucosal lymphoid tissue (RAMALT).

Cellular staining appears adequate, but cellular detail is not discernable due to poor scan (out of plane of focus on mucosal side)

The superficial mucosal epithelium is mostly intact; there are limited multifocal areas of lost surface columnar epithelium. Integrity is good. Multifocally the top 1/3 of surface epithelium is disorganized or lost.

In the squamous epithelium there is mild intracellular vacuolation of squamous

epithelial cells (intracellular edema).

Poor scan – blurry @ more than 10x on mucosal side of slide due to being out of plane of focus? Squamous side is ok

Project ID# 707: Kudva IVOC 030123 NB-B

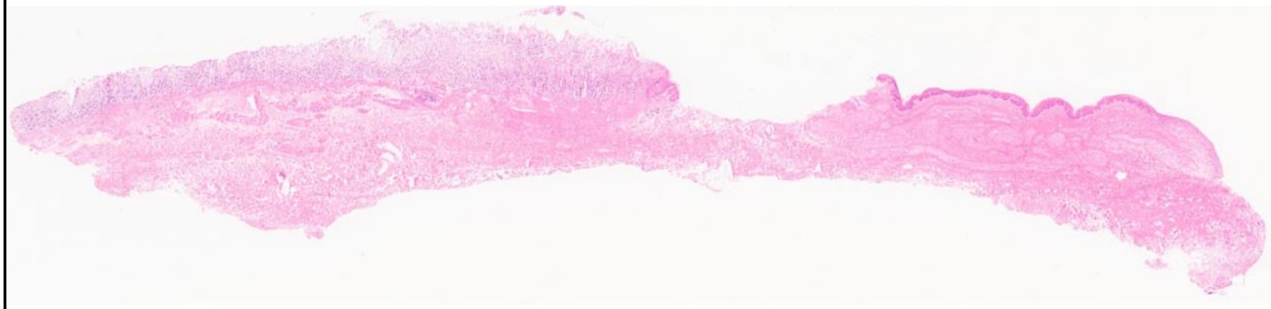

H&E-stained frozen section of recto-anal junction that contains squamous epithelium and mucosal columnar epithelial cells with abundant goblet cells. The mucosal portion of tissue contains mucosa, submucosa, circular smooth muscle of the tunic muscularis, and tunica adventitia. The oral 1/3 of the squamous epithelium is missing.

Within the mucosa and submucosa, there is a small single lymphoid nodule – recto-anal mucosal lymphoid tissue (RAMALT).

Cellular staining is good.

The superficial mucosal epithelium is intact in 30%. There is disruption/disorganization/ sloughing of the top 1/3 of mucosal surface in the other portions of mucosa. In the squamous epithelium there is mild intercellular bridging (intercellular edema) and intracellular vacuolation of squamous epithelial cells (intracellular edema).

There is a minimal infiltrate of inflammatory cells (lymphocytes and plasma cells) in the deep mucosa and lamina propria – likely native/ resident population. There is mild crypt dropout and an increased amount of fibrous connective tissue in the lamina propria.

Project ID# 707: Kudva IVOC 030123
EDL 932-B

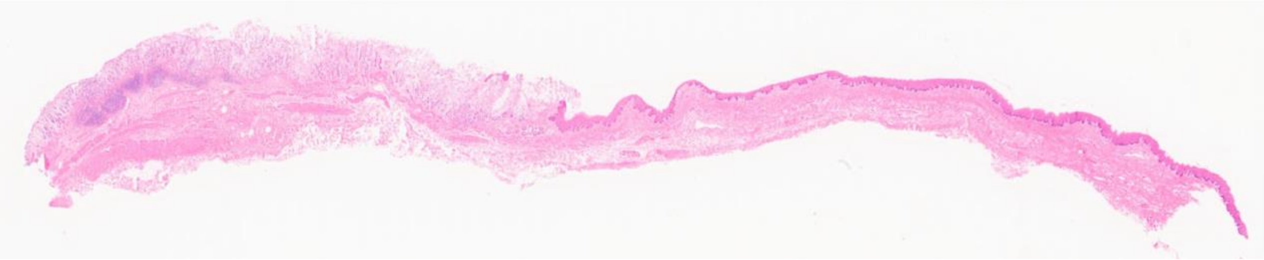

H&E-stained frozen section of recto-anal junction that contains squamous epithelium and mucosal columnar epithelial cells with abundant goblet cells. The mucosal portion of tissue contains mucosa, submucosa, circular smooth muscle of the tunic muscularis, and tunica adventitia.

Within the mucosa and submucosa, there 4-5 moderate sized lymphoid nodules – recto-anal mucosal lymphoid tissue (RAMALT).

Cellular staining is good.

The superficial mucosal epithelium is intact in 30%. There is disruption/disorganization/ sloughing of the top 1/3 of mucosal surface in the other portions of mucosa (closer to the RAJ). In the squamous epithelium there is mild intercellular bridging (intercellular edema) and intracellular vacuolation of squamous epithelial cells (intracellular edema).

There is a mild to moderate infiltrate of inflammatory cells (lymphocytes and plasma cells

and histiocytes) in mucosal lamina propria. There is mild crypt dropout and focall a dilated crypt (4-5x normal width)

Project ID# 707: Kudva IVOC 030123
EDL 932 ΔSLP-A

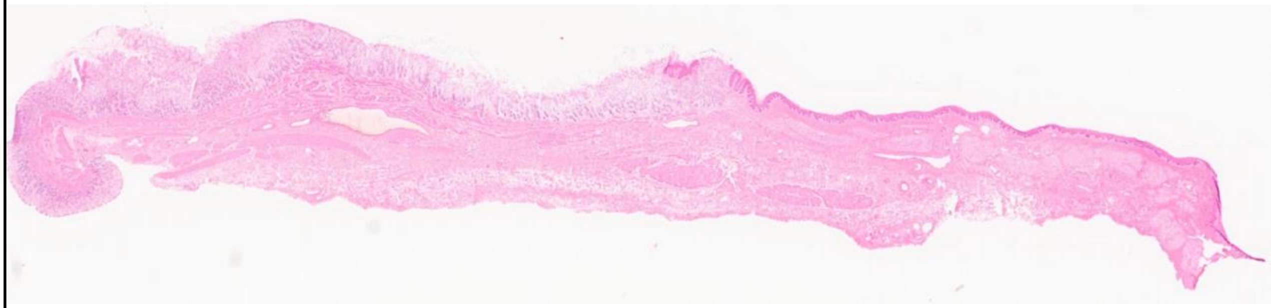

H&E-stained frozen section of recto-anal junction that contains squamous epithelium and mucosal columnar epithelial cells with abundant goblet cells. The mucosal portion of tissue contains mucosa, submucosa, circular smooth muscle of the tunic muscularis, and tunica adventitia.

Within the mucosa and submucosa, lymphoid nodules are absent – recto-anal mucosal lymphoid tissue (RAMALT).

Cellular staining is good.

The superficial mucosal epithelium is intact in 30%. There is disruption/disorganization/ sloughing of the top 1/3 of mucosal surface in the other portions of mucosa. In the squamous epithelium there is intracellular vacuolation of squamous epithelial cells (intracellular edema).

There is a mild to moderate infiltrate of inflammatory cells (lymphocytes and plasma cells and histiocytes) in the deep mucosa and lamina propria –. There is mild crypt dropout and

an increased amount of fibrous connective tissue in the lamina propria.

Project ID# 707: Kudva IVOC 030123
EDL 932 ΔSLP-A

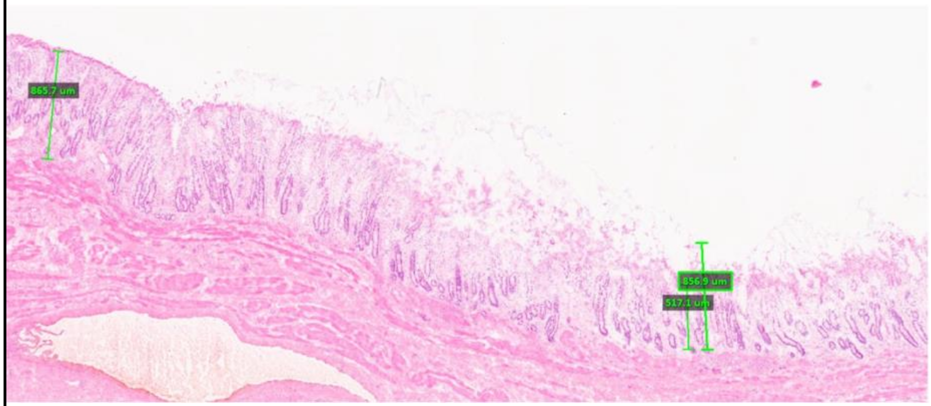

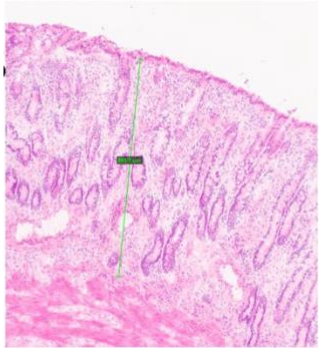

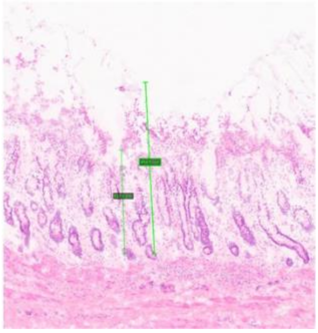

Example of disrupted/ sloughed superficial 1/3 of mucosal surface. Bottom 2/3 of mucosal maintains excellent structure and staining characteristics

Project ID# 707: Kudva IVOC 030123
EDL 932 ΔSLP-B

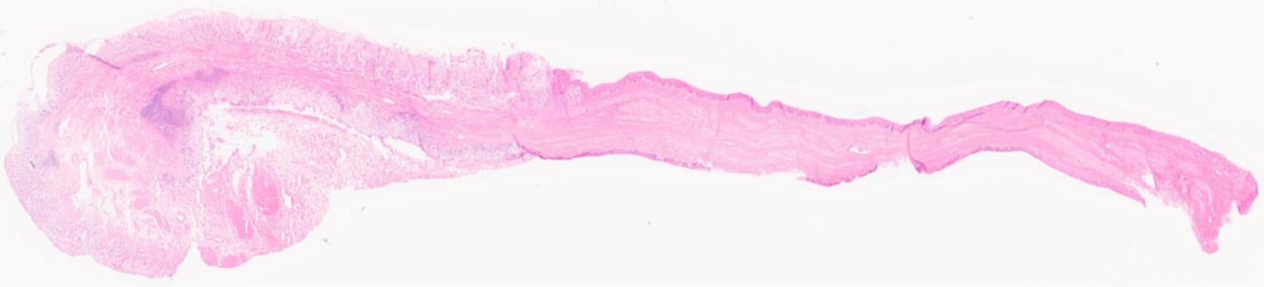

H&E-stained frozen section of recto-anal junction that contains squamous epithelium and mucosal columnar epithelial cells with abundant goblet cells. The mucosal portion of tissue contains mucosa, submucosa, circular smooth muscle of the tunic muscularis, and tunica adventitia. The section contains mucosa and squamous epithelium on the top and bottom – likely from embedding a curled section.

Within the mucosa and submucosa, there are a few lymphoid nodules – recto-anal mucosal lymphoid tissue (RAMALT).

Cellular staining is good.

The superficial mucosal epithelium is intact in ~20% of the section. The mucosal structural integrity is good; multifocally the top 1/3 of surface epithelium is disorganized or lost.

There is a mild infiltrate of inflammatory cells (lymphocytes and plasma cells and

histiocytes) in the mucosal lamina propria –. There is mild crypt dropout and an increased amount of fibrous connective tissue in the lamina propria.

In the squamous epithelium there is mild intracellular vacuolation of squamous epithelial cells (intracellular edema).

Project ID# 707: Kudva IVOC 030123
EDL 932 COMP-A

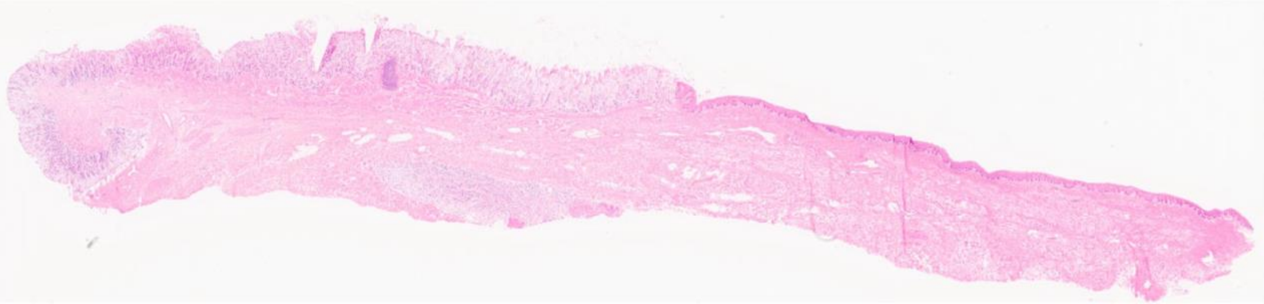

H&E-stained frozen section of recto-anal junction that contains squamous epithelium and mucosal columnar epithelial cells with abundant goblet cells. The mucosal portion of tissue contains mucosa, submucosa, circular smooth muscle of the tunic muscularis, and tunica adventitia. The section is slightly tangential and contains some mucosa on both sides (top and bottom)

Within the mucosa and submucosa, there is a single lymphoid nodule – recto-anal mucosal lymphoid tissue (RAMALT).

Cellular staining is good.

The superficial mucosal epithelium is intact in ~10-20% of the section. The mucosal structural integrity is good; multifocally the top 1/3 of surface epithelium is disorganized or lost.

There is a mild infiltrate of inflammatory cells (lymphocytes and plasma cells and histiocytes) in the mucosal lamina propria –. There is mild crypt dropout and an increased

amount of fibrous connective tissue in the lamina propria.

In the squamous epithelium there is mild intracellular vacuolation of squamous epithelial cells (intracellular edema).

Project ID# 707: Kudva IVOC 030123
EDL 932 COMP-B

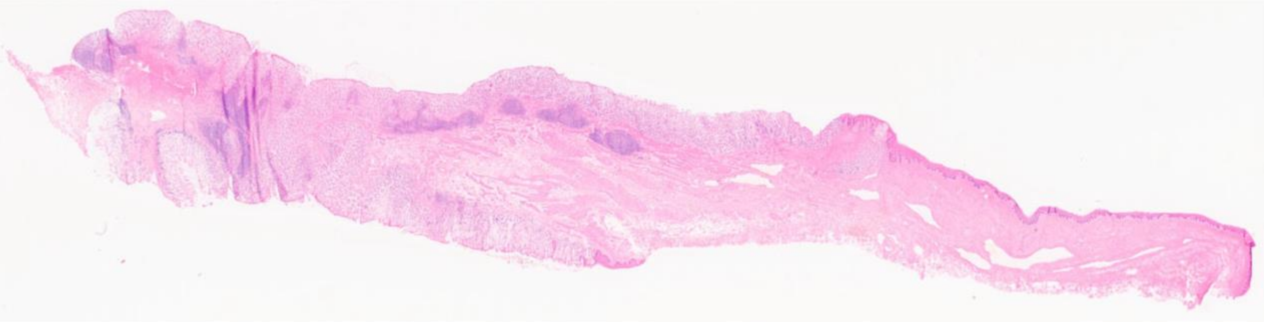

H&E-stained frozen section of recto-anal junction that contains squamous epithelium and mucosal columnar epithelial cells with abundant goblet cells. The mucosal portion of tissue contains mucosa, submucosa, circular smooth muscle of the tunic muscularis, and tunica adventitia. There are several folds in the tissue and the orientation of the section is not longitudinal – Section of tissue may be curled in paraffin.

Within the mucosa and submucosa, there are several lymphoid nodules – recto-anal mucosal lymphoid tissue (RAMALT).

Cellular staining is generally good; although; there is a focal limited area where nuclear/chromatin material is degrading.

The superficial mucosal epithelium is intact in >80%. In the squamous epithelium there is intracellular vacuolation of squamous epithelial cells (intracellular edema).

There is a mild to moderate infiltrate of inflammatory cells (lymphocytes and plasma cells and histiocytes) in the deep mucosa and lamina propria –. Fibrous connective tissue in the lamina propria is prominent (mild increase)

Project ID# 707: Kudva IVOC 030123
EDL 932 COMP-B

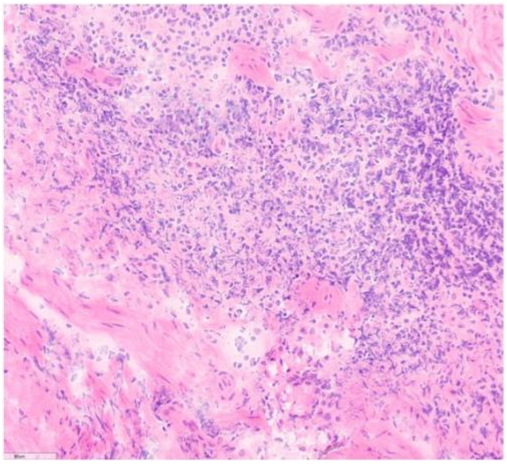

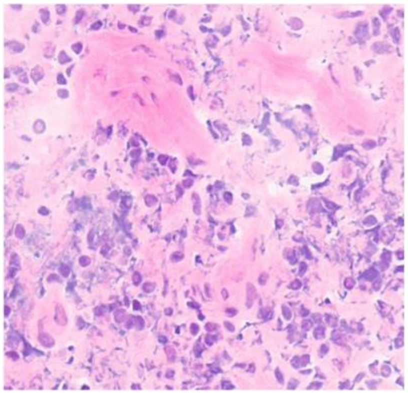

Degradation and smearing of deeply basophilic material (nuclear contents/ chromatin) – consistent with crush artifact – i.e. forceps

Overall, cellular integrity is still good in the section.

Project ID# 707: Kudva IVOC 030123
EDL 932-A

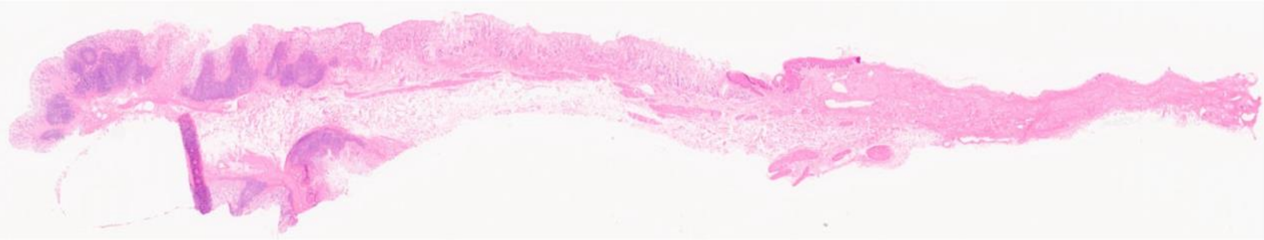

H&E-stained frozen section of recto-anal junction that contains squamous epithelium and mucosal columnar epithelial cells with abundant goblet cells. The mucosal portion of tissue contains mucosa, submucosa, circular smooth muscle of the tunic muscularis, and tunica adventitia. There is a fold in the tissue and the orientation of the section indicates that the tissue may be curled in paraffin. 75% of the squamous epithelium is absent.

Within the mucosa and submucosa, there are several lymphoid nodules – recto-anal mucosal lymphoid tissue (RAMALT).

Cellular staining is generally good.

The superficial mucosal epithelium is intact in ~20%. In the squamous epithelium there is intracellular vacuolation of squamous epithelial cells (intracellular edema).

There is a mild infiltrate of inflammatory cells (lymphocytes and plasma cells and histiocytes) in the deep mucosa and lamina propria. Fibrous connective tissue in the lamina

propria is prominent (mild increase).

Summary

Overall, the cellular staining and tissue integrity of the IVOC was good to excellent. Some disruption of superficial mucosal epithelium and lamina proprio was observed in some subsets/ groups. The Pre-assay had the best histologic preservation.
Differences were seen between IDs.

Project ID# 703: Kudva IVOC 011123

Samples had inflammatory infiltrates suggesting chronic antigenic stimulation – antemortem.

Project ID# 704: Kudva IVOC 012623

Samples had antemortem granulomatous lymphoplasmacytic proctitis/colitis and lymphoid hyperplasia. The presence of epithelioid macrophages and multinucleated cells is consistent with Johne's disease. Many Mott Cells suggesting chronic antigenic stimulation

Project ID# 707: Kudva IVOC 030123

Limited GALT. Suggesting possible an older animal or an animal housed indoors in containment (lacking normal levels environmental antigenic stimulation)
