# Supplementary material for: The carbon starvation-inducible lipoprotein (Slp) influences differential adherence of Escherichia coli O157:H7 at the bovine rectoanal junction
Source: PLoS Pathog. 2026 May 18;22(5):e1013584. doi: 10.1371/journal.ppat.1013584 (PMC13193606; doi:10.1371/journal.ppat.1013584)

Histopathology Results
IVOC samples, 7-22-21 and 7-29-21
NBA, NBB, NBC, NBD, EDL932A and B, EDL932 SLP A and B, EDL932 comp A and B

by
Dr. Mitchell Palmer

8-26-2021

Summary

- All NB samples of rectoanal junction have both squamous and glandular portions present. All have mucosa that is normal in appearance with intact epithelium.
- The primary and only change in all other specimens was mucosal disruption of the glandular region epithelium to varying degrees.
- Squamous regions on all slides were unremarkable.
- Samples EDL932 comp A had small colonies of short bacilli or mixed bacterial morphology associated with mucus or other debris, which was present near or attached to the mucosal surface.

NB A and B
7-22-21 and 7-29-21

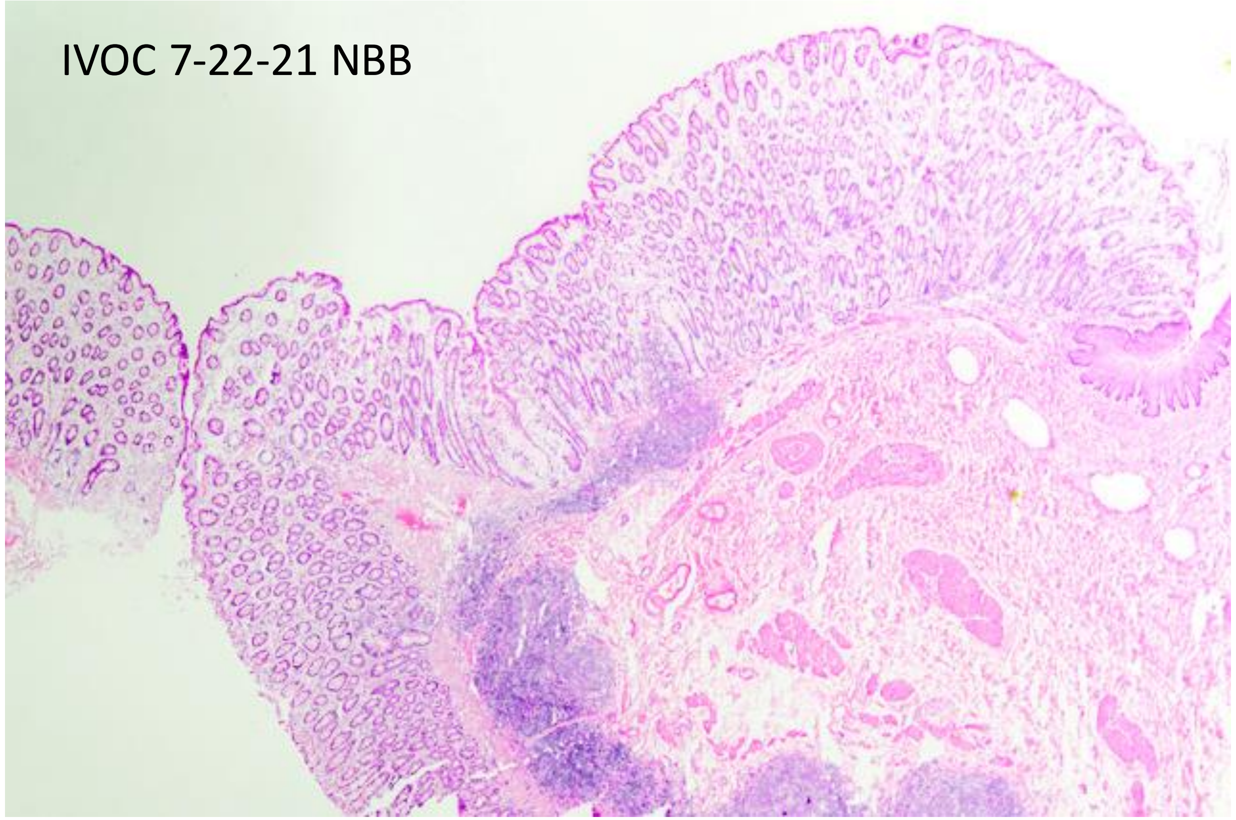

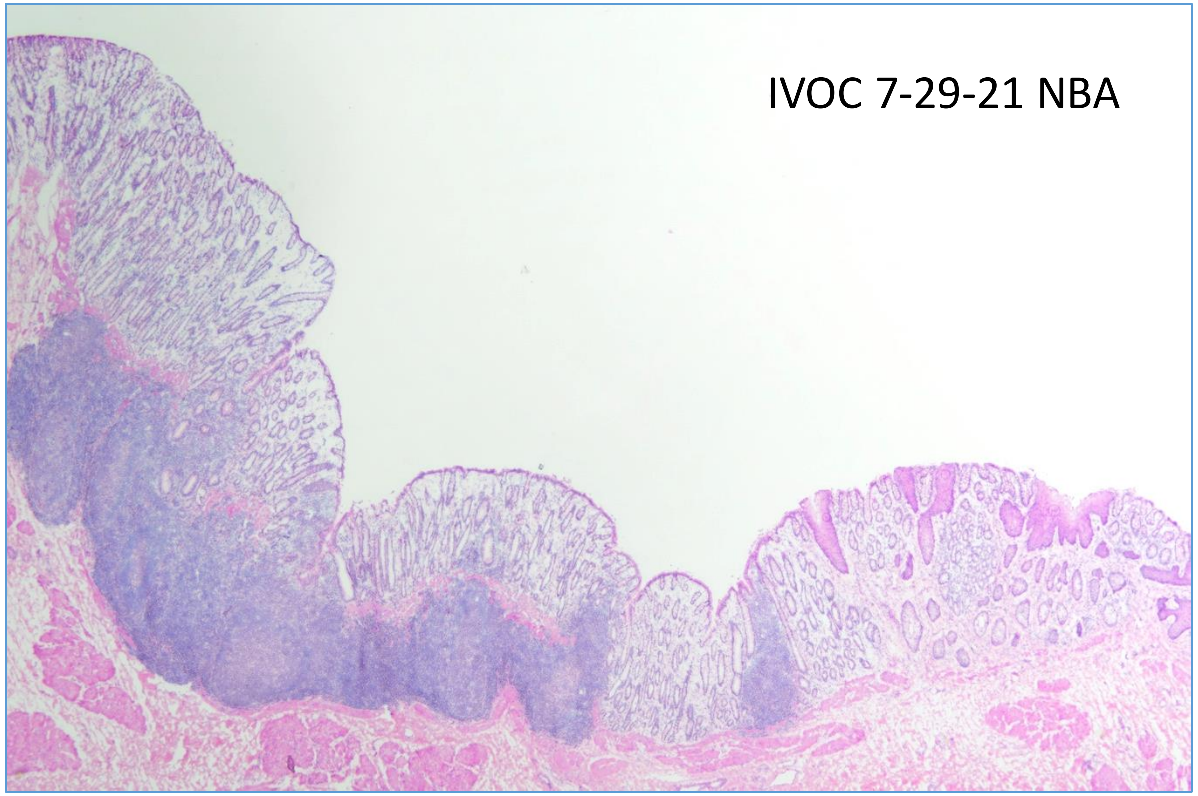

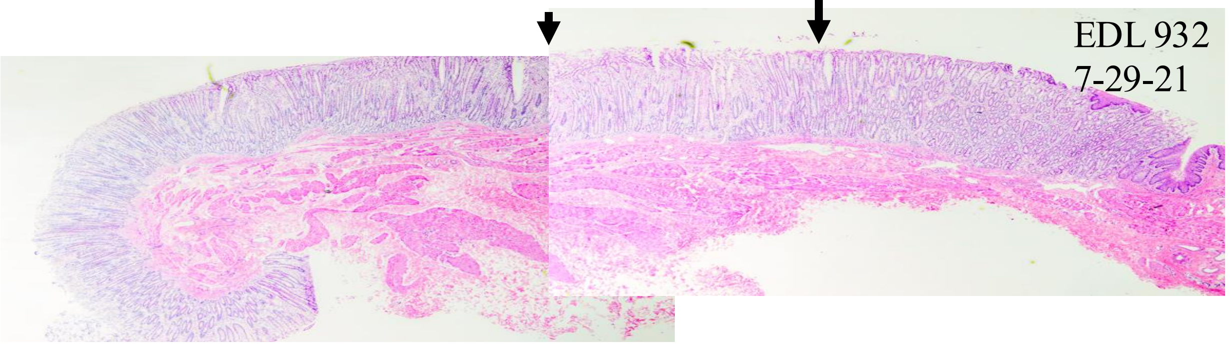

This is a section of rectoanal junction with remnants of both glandular and squamous portions present. There is superficial mucosal disruption over approx. 30% of the section (arrows). The remainder of the epithelium and squamous regions are unremarkable.

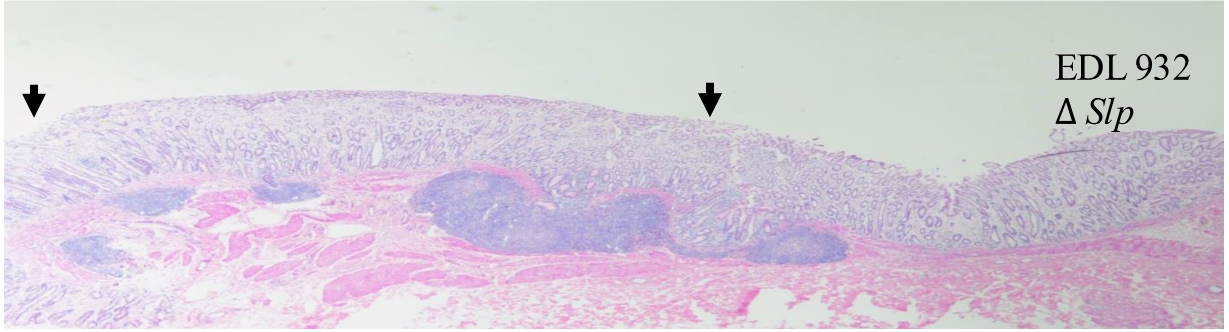

This is a section of rectoanal junction with remnants of both glandular and squamous portions present. There are 2 small foci of superficial mucosal disruption (arrows). The remainder of the epithelium and squamous regions are unremarkable.

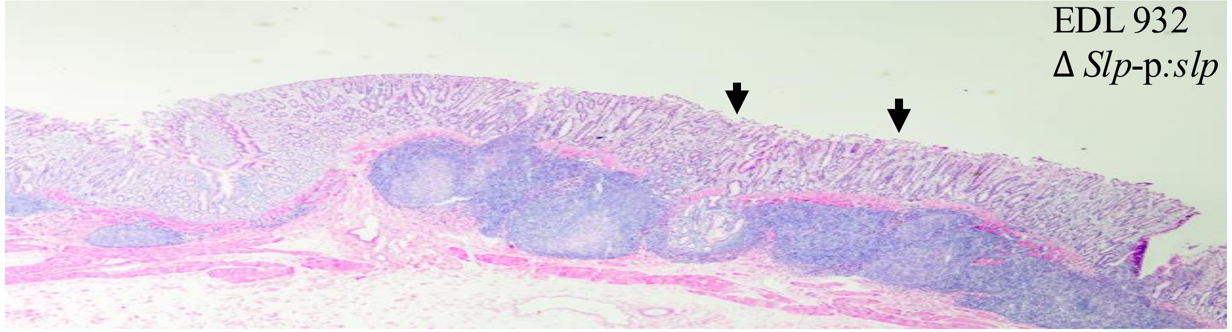

This is a section of rectoanal junction with remnants of both glandular and squamous portions present. There is superficial mucosal disruption over approx. 5% of the section (arrows). The remainder of the epithelium and squamous regions are unremarkable.

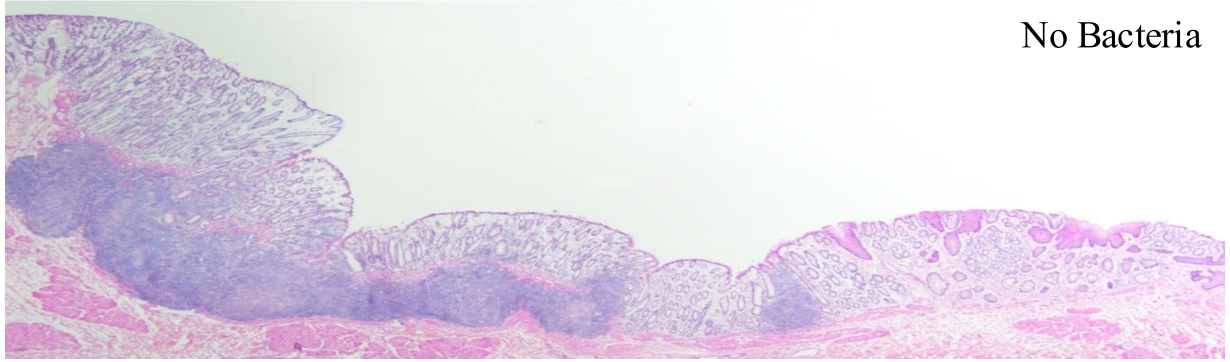

These are sections of rectoanal junction with both glandular and squamous regions present. The glandular mucosa is intact. The squamous portion is unremarkable.

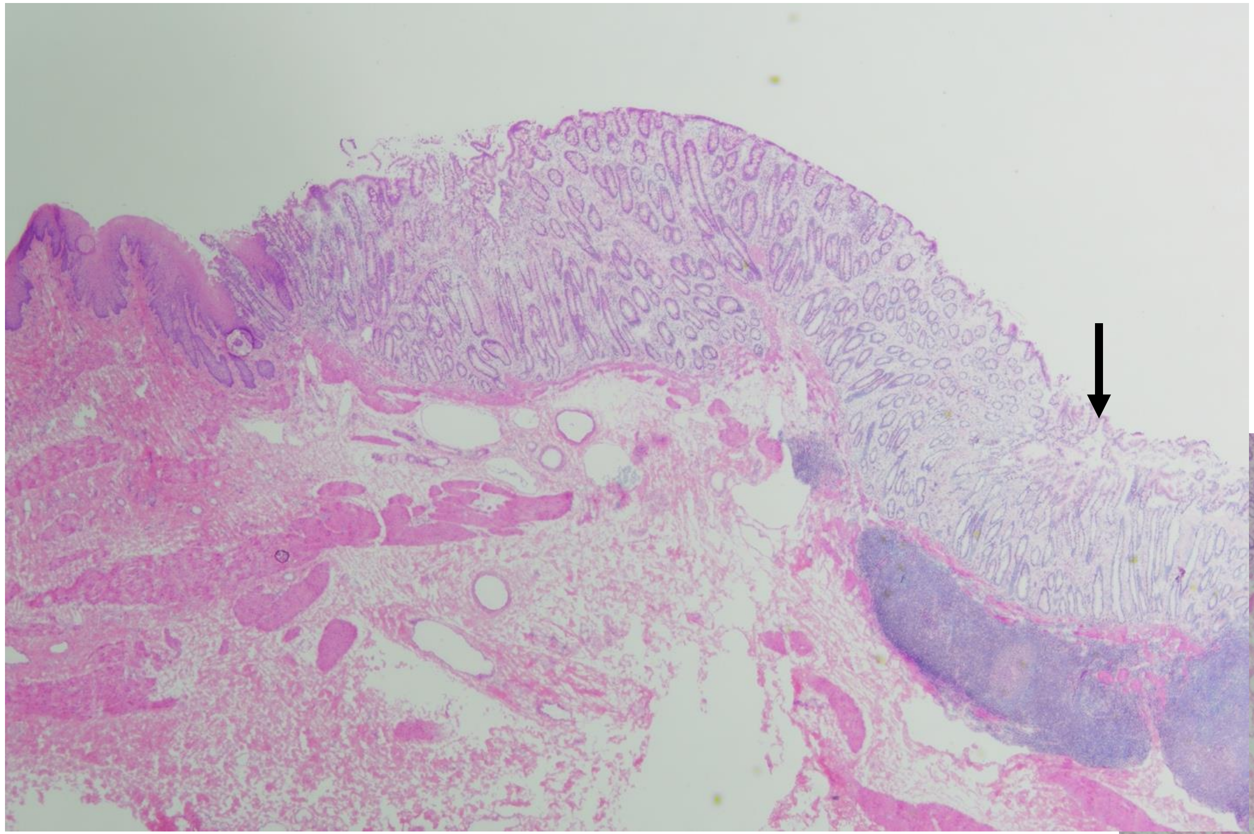

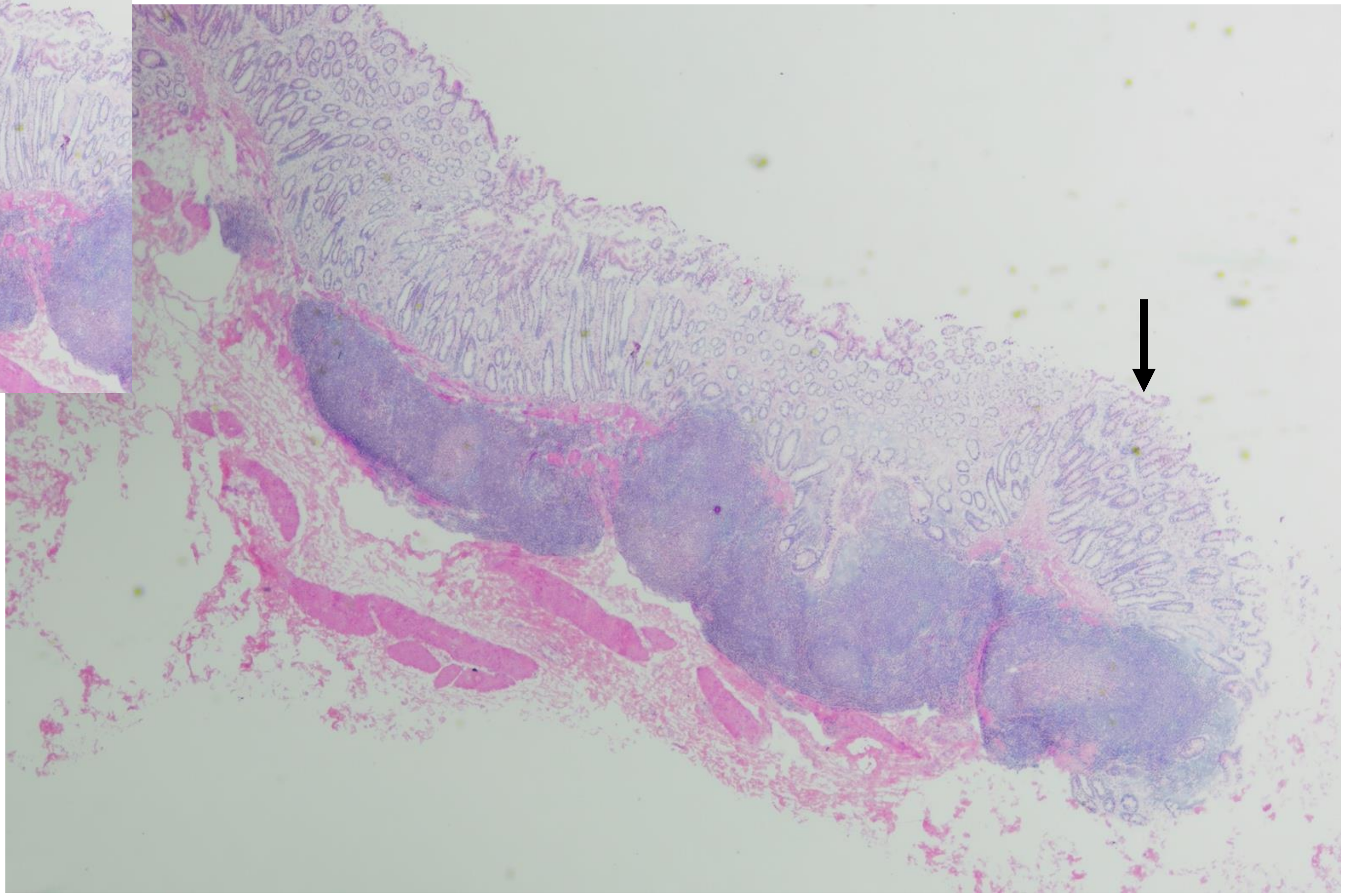

EDL 932
7-22-21

EDL932 SLP
7-22-21

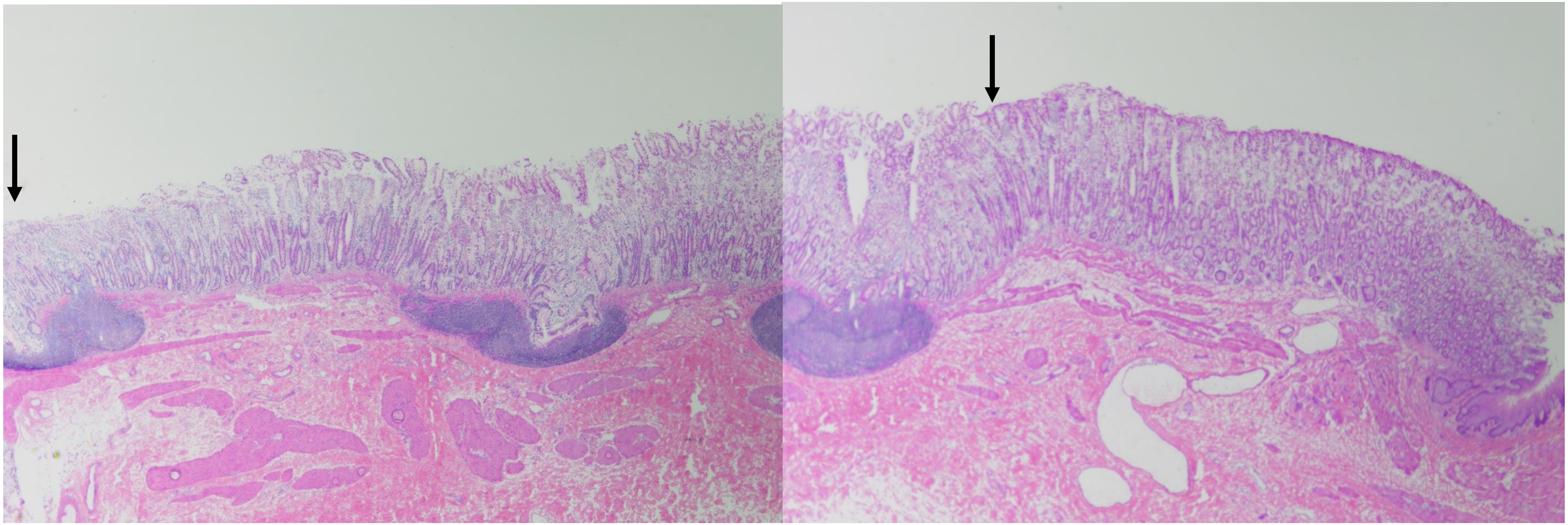

EDL 932 comp
7-22-21

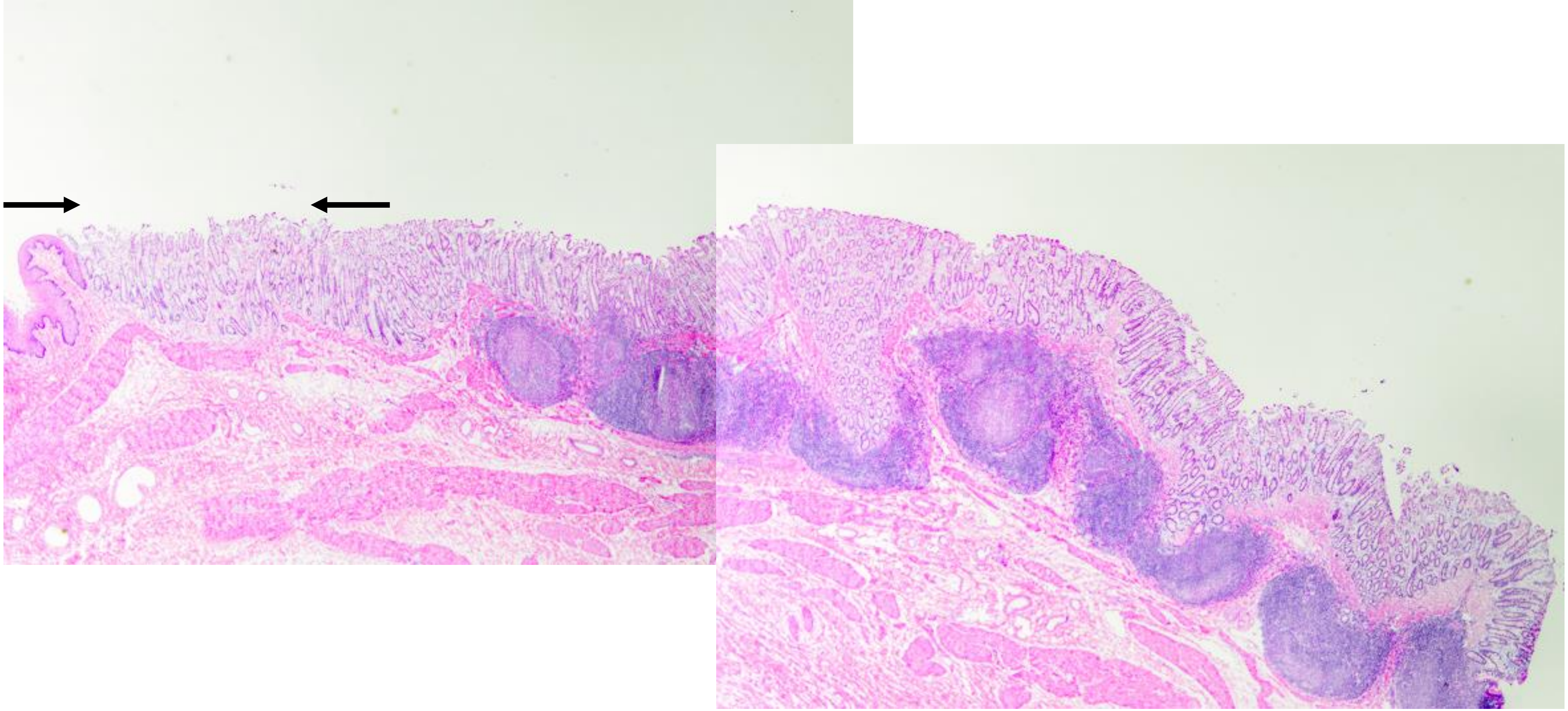

EDL 932 A
7-29-21

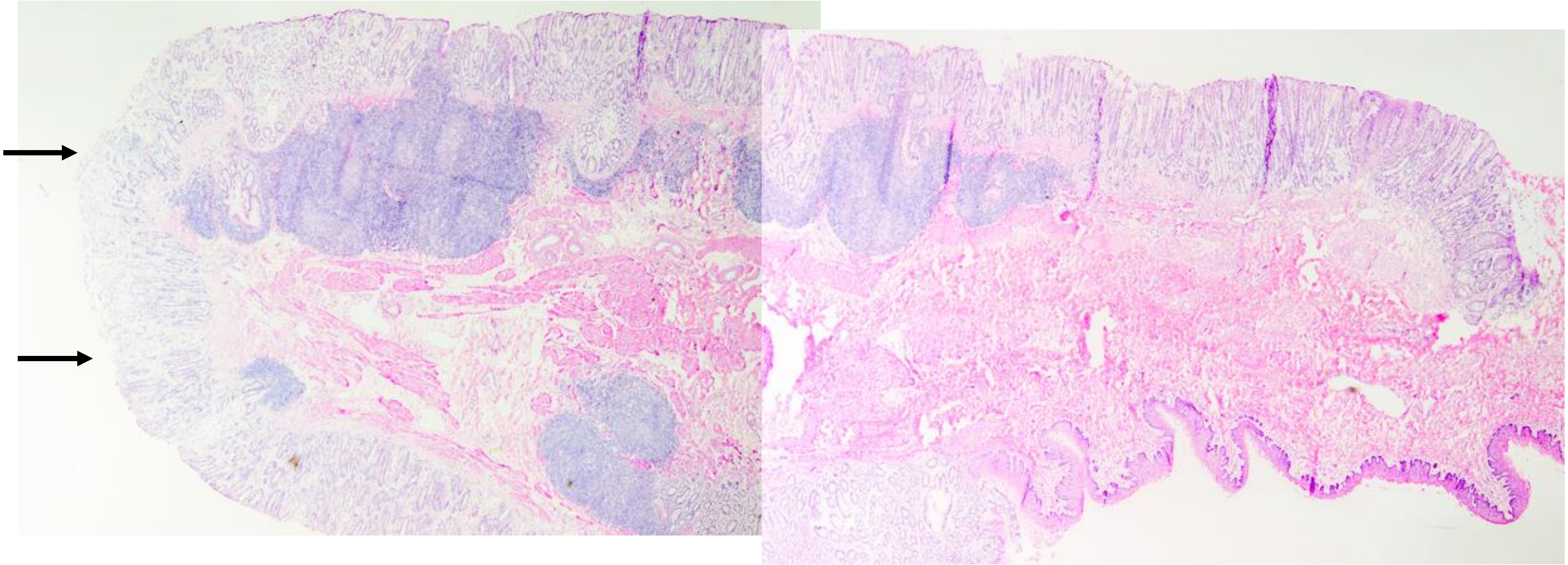

EDL 932 B
7-29-21

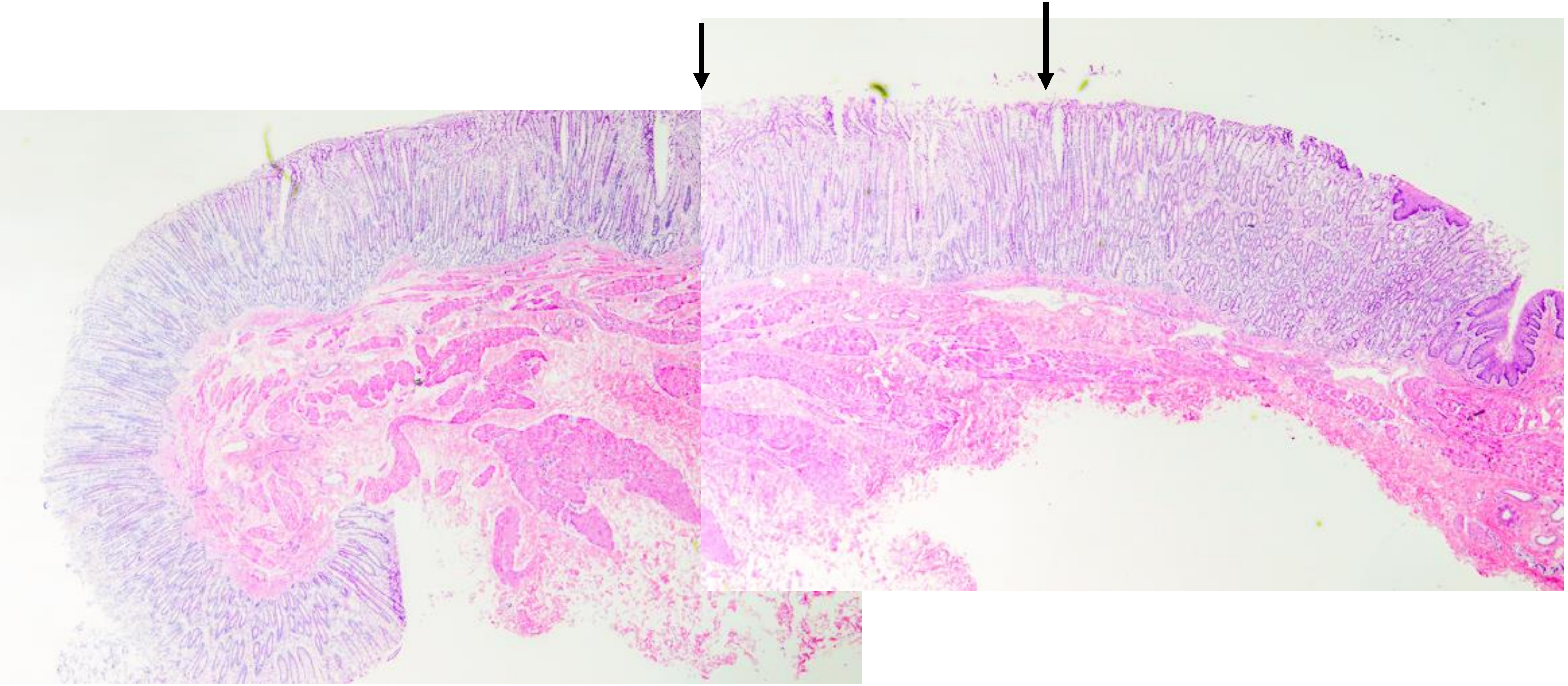

EDL 932 SLP A
7-29-21

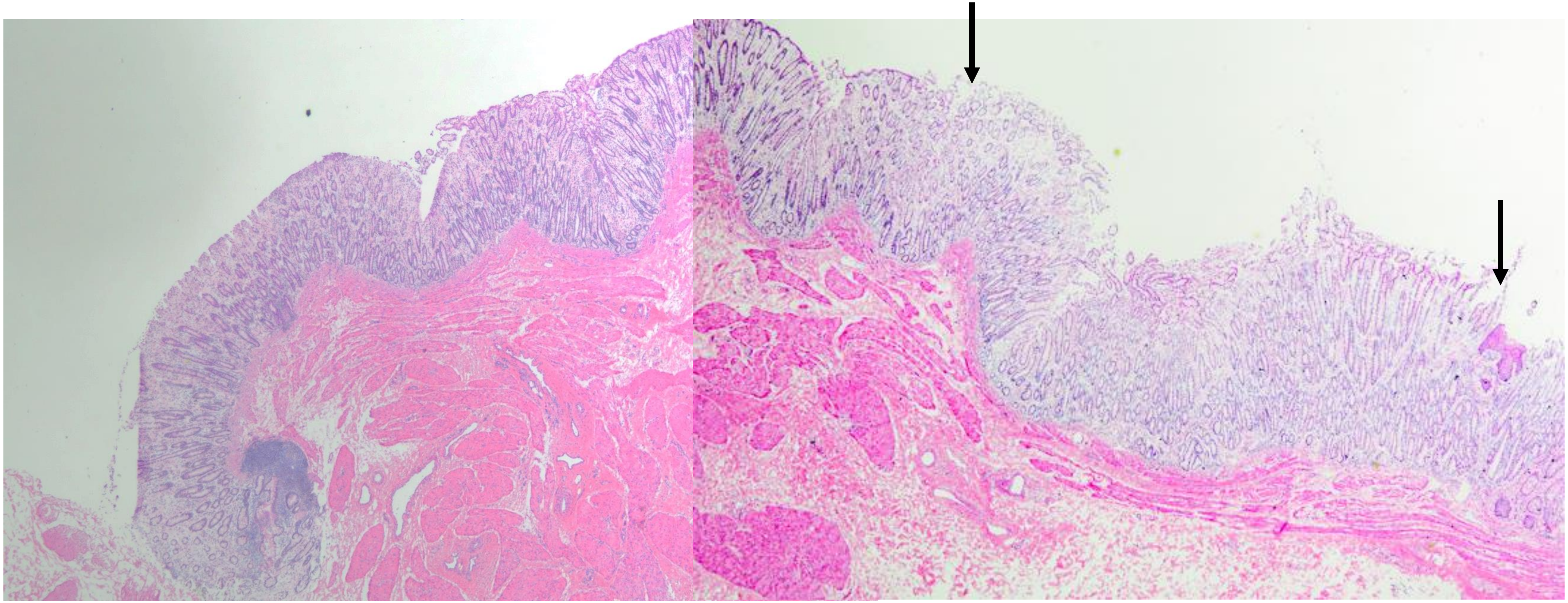

EDL 932 SLP B
7-29-21

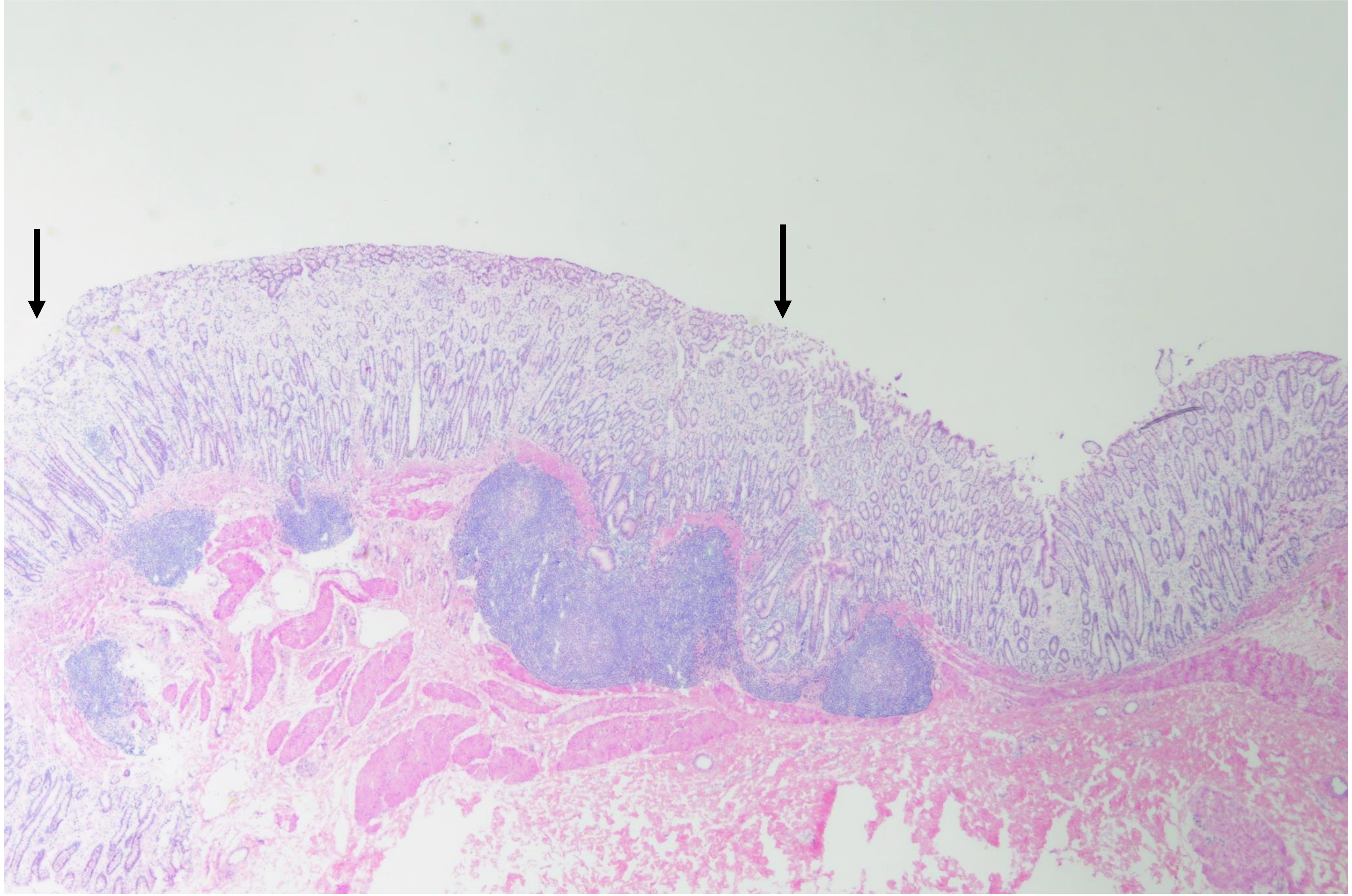

EDL 932 COMP A
7-29-21

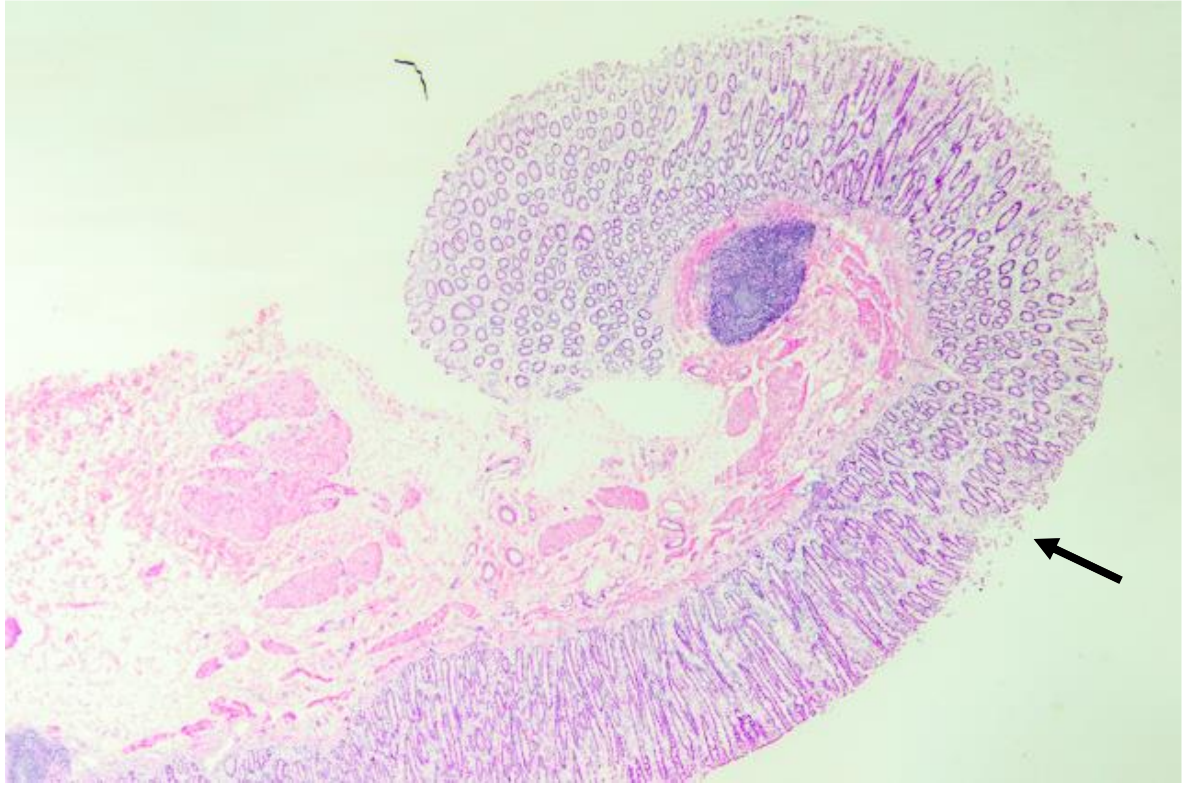

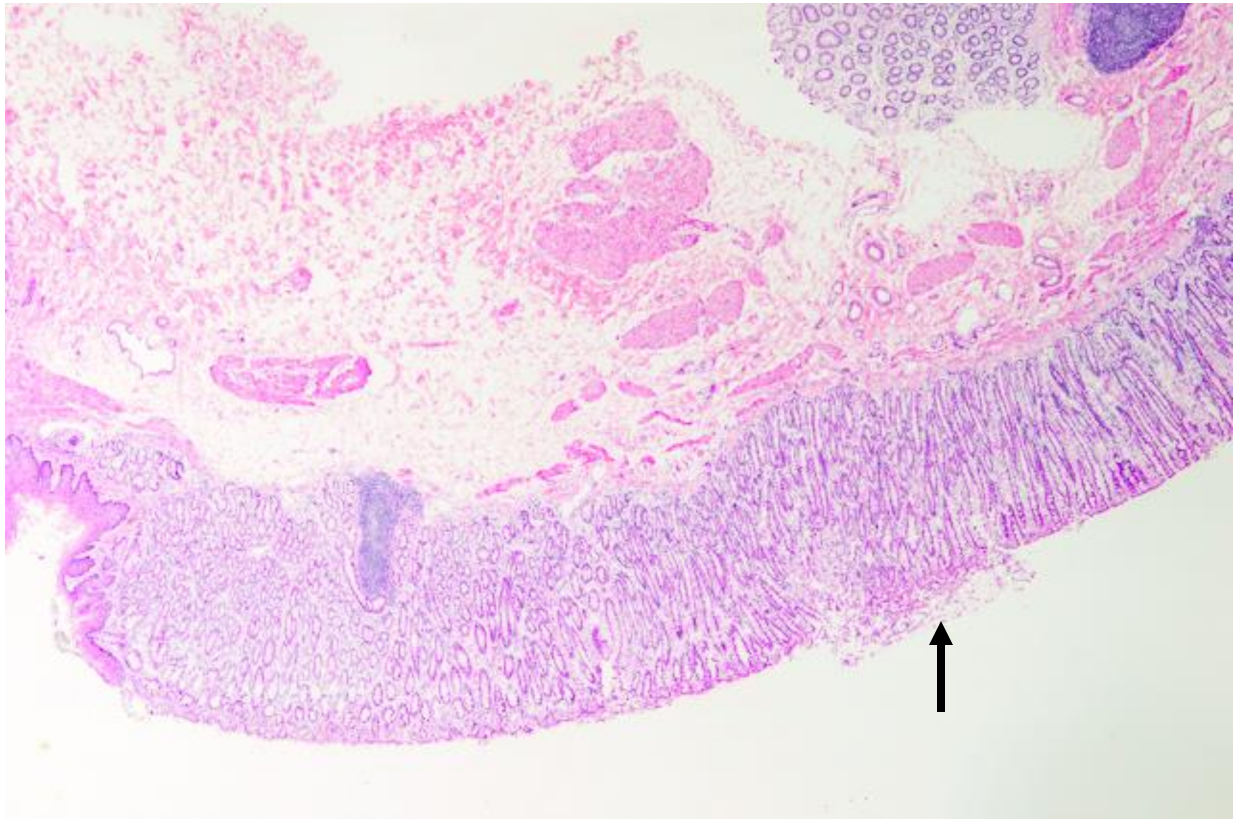

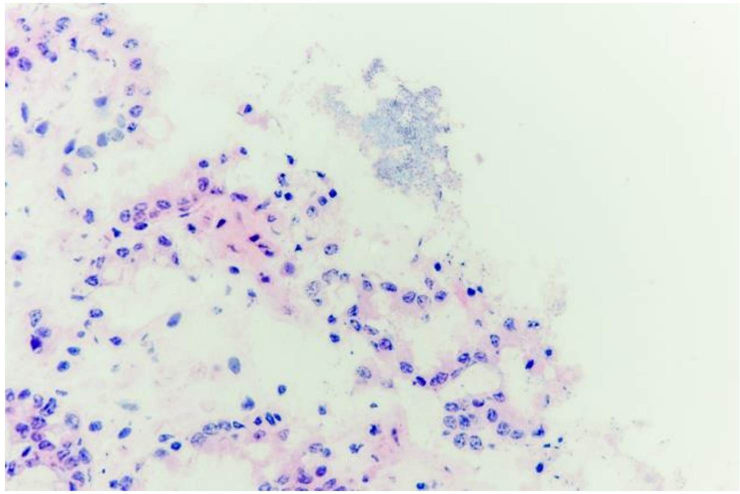

EDL 932 COMP B
7-29-21

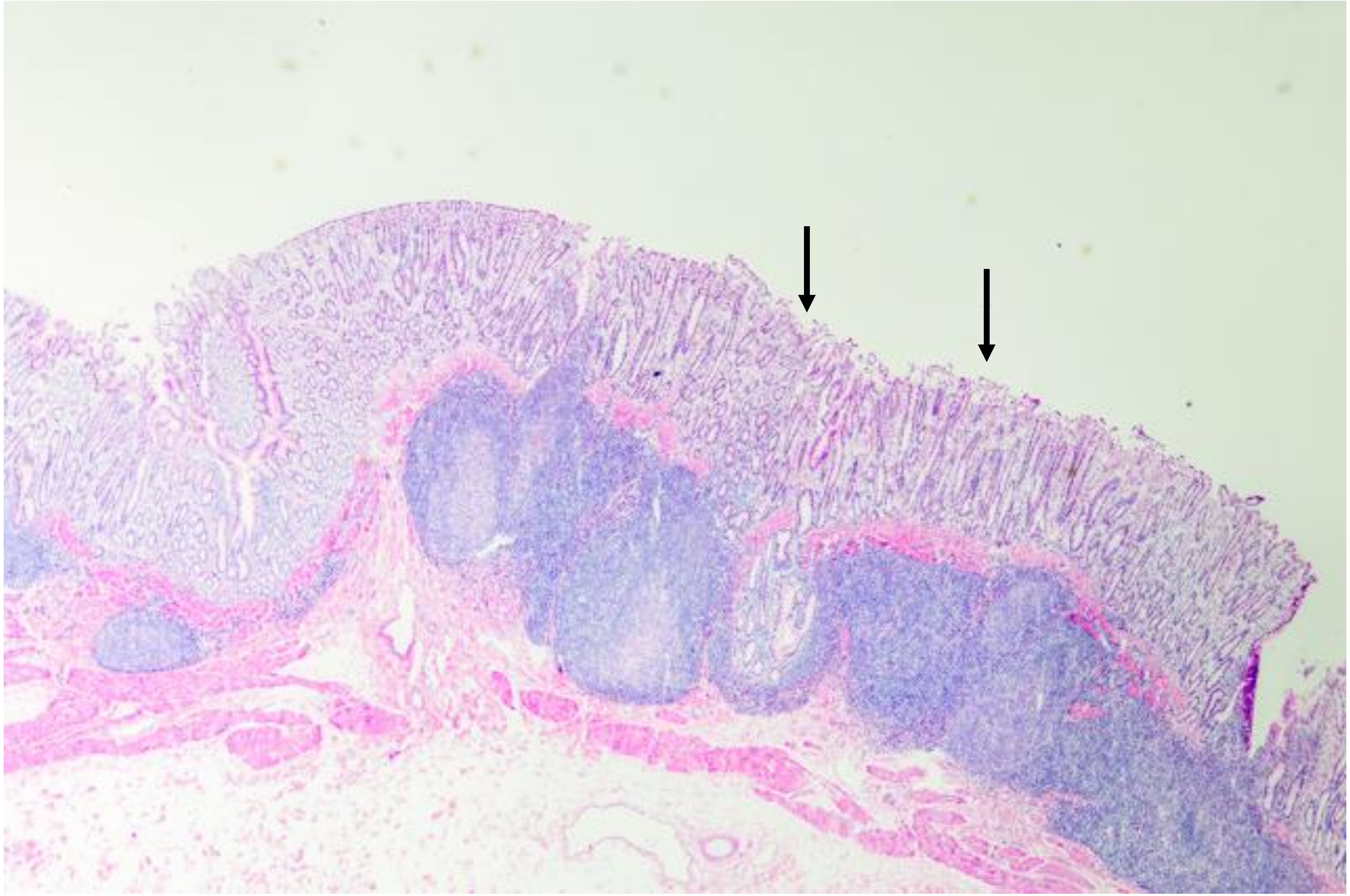

Supplement: S1 Data — (ZIP) [file ppat.1013584.s010.zip › S1_Data/PalmerResults_IVOC 7-21-21 and 7-29-21.pdf]
