# Supplementary material for: The carbon starvation-inducible lipoprotein (Slp) influences differential adherence of Escherichia coli O157:H7 at the bovine rectoanal junction
Source: PLoS Pathog. 2026 May 18;22(5):e1013584. doi: 10.1371/journal.ppat.1013584 (PMC13193606; doi:10.1371/journal.ppat.1013584)
Supplement: S4 Data — (DOCX) [file ppat.1013584.s013.docx]

**BLASTp Results:** Bovine pIGR vs/ Human pIGR

Query: Bovine pIGR; Length: 757

Subject: Human pIGR Length: 764

Range 1: 1 to 764

Identities:510/765(67%), Positives:603/765(78%), Gaps:9/765(1%)


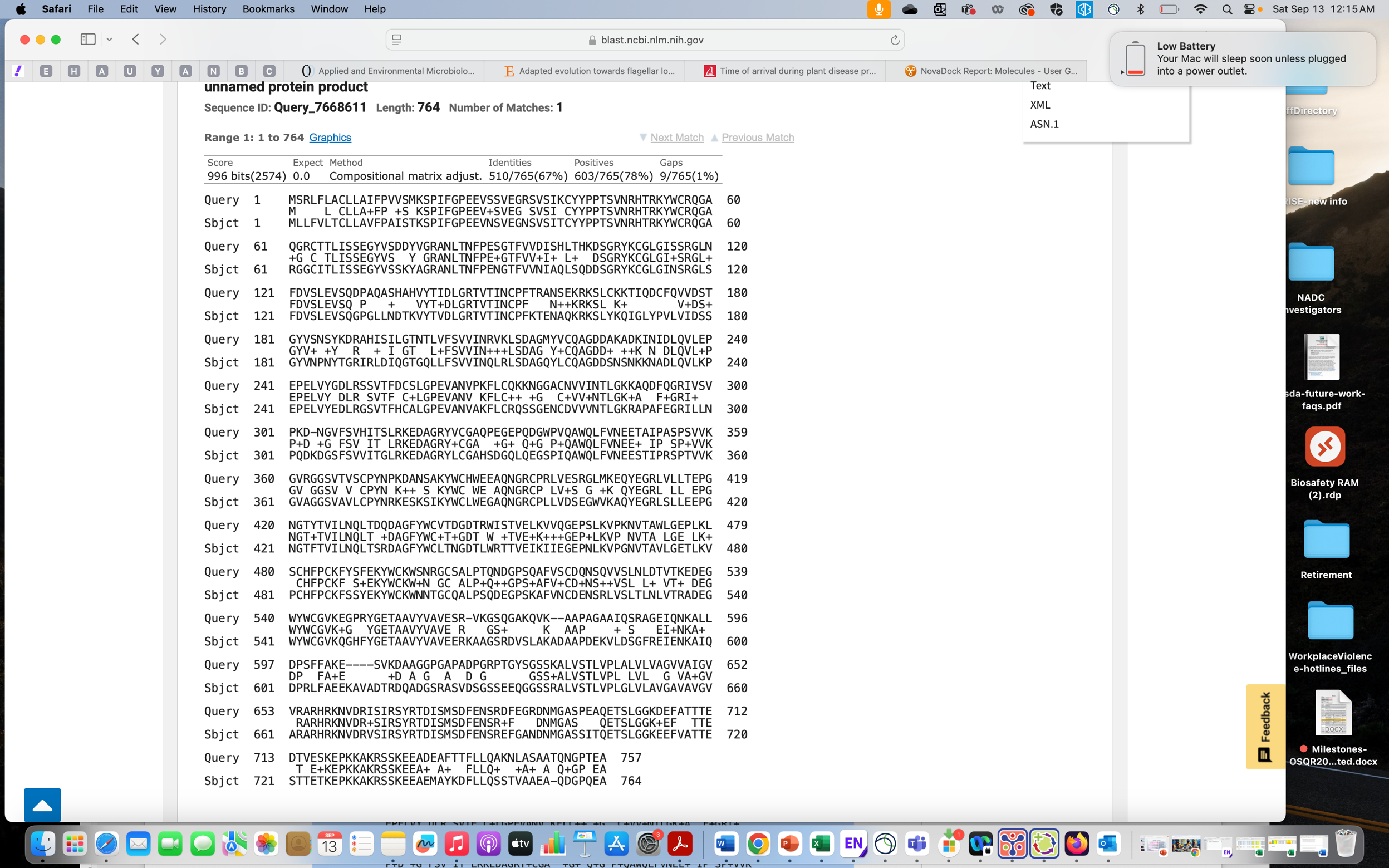


**MODEL Information**

**Bovine pIGR-Slp Model 1**

**No filtering**

**Molecule:** *Model 1*

**Energy:** -34.95

**Cluster size:** 1

**Cluster energy:** -34.95 ± 0.00

**Residue contacts:** 41 (The number of ligand residues in contact with receptor residues in a particular model).

Residue Molecule Intermolecular Contacts

true A:PHE 13 Receptor a:GLU 167

true A:MET 18 Receptor a:TRP 170

true A:GLY 611 Receptor a:GLU 148

true A:GLY 612 Receptor a:GLU 148

true A:PRO 613 Receptor a:ARG 147, a:GLU 148

true A:GLY 646 Receptor a:ALA 163

true A:ILE 650 Receptor a:ALA 163, a:GLY 162, a:TRP 165, a:TYR 161

true A:VAL 652 Receptor a:GLU 167

true A:VAL 653 Receptor a:GLU 167, a:PRO 166, a:PRO 168, a:TRP 165

true A:ARG 654 Receptor a:GLY 160, a:TRP 165, a:TYR 159, a:TYR 161

true A:ARG 656 Receptor a:GLU 167, a:PRO 168

true A:HIS 657 Receptor a:ALA 172, a:PRO 168, a:PRO 173, a:TYR 159, a:TYR 175

true A:ASN 660 Receptor a:ALA 172, a:PRO 168, a:TRP 170

true A:VAL 661 Receptor a:ALA 172, a:TYR 175

true A:ILE 664 Receptor a:ALA 172, a:PRO 173, a:TYR 174, a:TYR

true A:ILE 666 Receptor a:TYR 174, a:TYR 175

true A:ARG 670 Receptor a:GLU 148

true A:LYS 728 Receptor a:ALA 178, a:THR 176

true a:ARG 147 Ligand A:PRO 613

true a:GLU 148 Ligand A:ARG 670, A:GLY 611, A:GLY 612, A:PRO 613

true a:TYR 159 Ligand A:ARG 654, A:HIS 657

true a:GLY 160 Ligand A:ARG 654

true a:TYR 161 Ligand A:ARG 654, A:ILE 650

true a:GLY 162 Ligand A:ILE 650

true a:ALA 163 Ligand A:GLY 646, A:ILE 650

true a:TRP 165 Ligand A:ARG 654, A:ILE 650, A:VAL 653

true a:PRO 166 Ligand A:VAL 653

true a:GLU 167 Ligand A:ARG 656, A:PHE 13, A:VAL 652, A:VAL 653

true a:PRO 168 Ligand A:ARG 656, A:ASN 660, A:HIS 657, A:VAL 653

true a:TRP 170 Ligand A:ASN 660, A:MET 18

true a:ALA 172 Ligand A:ASN 660, A:HIS 657, A:ILE 664, A:VAL 661

true a:PRO 173 Ligand A:HIS 657, A:ILE 664

true a:TYR 174 Ligand A:ILE 664, A:ILE 666

true a:TYR 175 Ligand A:HIS 657, A:ILE 664, A:ILE 666, A:VAL 661

true a:THR 176 Ligand A:LYS 728

true a:ALA 178 Ligand A:LYS 728

**Human pIGR-Slp Model 1**

**Filter:** Remove if not in binding funnel

**Molecule:** *Model 1*

**Energy:** -18.30

**Cluster size:** 2

**Cluster energy:** -12.01 ± 6.29

**Residue contacts:** 40 (The number of ligand residues in contact with receptor residues in a particular model).

Residue Molecule Intermolecular Contacts

true A:HIS 50 Receptor a:GLN 44

true A:THR 51 Receptor a:GLN 44, a:LEU 47

true A:ARG 52 Receptor a:ALA 40, a:GLN 44, a:LEU 47, a:TYR 48

true A:ARG 61 Receptor a:ASN 30, a:PRO 32

true A:GLY 62 Receptor a:PRO 32

true A:GLY 63 Receptor a:PRO 32

true A:ILE 65 Receptor a:LYS 36

true A:GLU 71 Receptor a:ALA 40, a:ASN 43, a:GLN 44, a:VAL 39

true A:GLY 72 Receptor a:VAL 39

true A:TYR 73 Receptor a:ALA 40, a:SER 37, a:VAL 39

true A:GLY 113 Receptor a:LEU 47

true A:ILE 114 Receptor a:GLN 35, a:GLN 51, a:LEU 47, a:TYR 48

true A:SER 116 Receptor a:ASP 33, a:GLN 35, a:GLN 51

true A:ARG 117 Receptor a:GLN 51, a:LEU 47

true A:ARG 675 Receptor a:GLY 46

true A:SER 676 Receptor a:GLY 46, a:LEU 47

true A:TYR 677 Receptor a:GLU 121, a:GLY 46

true A:ARG 678 Receptor a:ASN 132, a:GLU 121, a:PRO 123

true A:THR 679 Receptor a:ASN 43, a:GLN 44

true a:ASN 30 Ligand A:ARG 61

true a:PRO 32 Ligand A:ARG 61, A:GLY 62, A:GLY 63

true a:ASP 33 Ligand A:SER 116

true a:GLN 35 Ligand A:ILE 114, A:SER 116

true a:LYS 36 Ligand A:ILE 65

true a:SER 37 Ligand A:TYR 73

true a:VAL 39 Ligand A:GLU 71, A:GLY 72, A:TYR 73

true a:ALA 40 Ligand A:ARG 52, A:GLU 71, A:TYR 73

true a:ASN 43 Ligand A:GLU 71, A:THR 679

true a:GLN 44 Ligand A:ARG 52, A:GLU 71, A:HIS 50, A:THR 51, A:THR 679

true a:GLY 46 Ligand A:ARG 675, A:SER 676, A:TYR 677

true a:LEU 47 Ligand A:ARG 117, A:ARG 52, A:GLY 113, A:ILE 114, A:SER 676, A:THR 51

true a:TYR 48 Ligand A:ARG 52, A:ILE 114

true a:GLN 51 Ligand A:ARG 117, A:ILE 114, A:SER 116

true a:GLU 121 Ligand A:ARG 678, A:TYR 677

true a:PRO 123 Ligand A:ARG 678

true a:ASN 132 Ligand A:ARG 678
